# Accuracy of Medical Image–Based Deep Learning for Detecting Microvascular Invasion in Hepatocellular Carcinoma: Systematic Review and Meta-Analysis

**DOI:** 10.2196/82000

**Published:** 2026-03-02

**Authors:** Wei Feng, Bo Qu, Shuo Han

**Affiliations:** 1 Department of Ultrasound, the Fourth Affiliated Hospital, China Medical University, Shenyang, China; 2 Department of Urology, Jinqiu Hospital of Liaoning Province, Shenyang, China; 3 Department of Cardiology, the Fourth Affiliated Hospital, China Medical University, No. 4, Chongshan East Road, Huanggu District, Shenyang, 110032, China, 86 18900912036

**Keywords:** deep learning, hepatocellular carcinoma, medical imaging, microvascular invasion, artificial intelligence

## Abstract

**Background:**

Hepatocellular carcinoma (HCC) is a leading cause of cancer-related mortality worldwide. Microvascular invasion (MVI) is a critical pathological indicator of postoperative recurrence and poor prognosis in patients with HCC. Some researchers have explored the diagnostic accuracy of deep learning (DL) based on various imaging modalities for MVI.

**Objective:**

This meta-analysis aimed to systematically evaluate the preoperative diagnostic performance of DL models using medical images to predict MVI in HCC, and to investigate the impact of different imaging modalities and validation strategies on model performance and generalizability.

**Methods:**

PubMed, Cochrane Library, Embase, and Web of Science were searched up to October 16, 2025. Studies investigating the detection of MVI in HCC using imaging-based DL techniques were eligible. Studies focusing solely on image segmentation were excluded. The Quality Assessment of Diagnostic Accuracy Studies-2 tool was used to assess risk of bias. A bivariate mixed-effects meta-analysis was performed to calculate the pooled sensitivity, specificity, and area under the summary receiver operating characteristic curve (SROC). Subgroup analyses were conducted by imaging modality and validation set generation method.

**Results:**

This meta-analysis included 52 studies with 19,531 patients with HCC. The pooled analysis revealed that imaging-based DL models had an overall sensitivity of 0.80 (95% CI 0.78‐0.83), a specificity of 0.82 (95% CI 0.80‐0.85), and an SROC of 0.88 for MVI prediction. Subgroup analysis showed that models based on preoperative contrast-enhanced computed tomography performed excellently, with a sensitivity of 0.84 (95% CI 0.79‐0.88), a specificity of 0.83 (95% CI 0.77‐0.88), and an SROC of 0.90. These results suggest that contrast-enhanced computed tomography is the most promising noninvasive method for current clinical applications. Meanwhile, DL models using pathological sections achieved the highest diagnostic performance: a sensitivity of 0.91 (95% CI 0.87‐0.94), a specificity of 0.90 (95% CI 0.68‐0.97), and an SROC of 0.92. This establishes the ultimate benchmark for performance optimization for all noninvasive models. A key finding was that model performance was less consistent in independent external validation (SROC: 0.85) than in internal validation (SROC: 0.90). This discrepancy indicates that overreliance on internal validation may overestimate model efficacy and underscores the decisive role of rigorous external validation in assessing real-world generalizability.

**Conclusions:**

This study is the first to systematically assess the use of imaging-based DL for diagnosing MVI in HCC. The results demonstrate a significant potential for these models in predicting MVI. However, their clinical applicability requires rigorous evaluation, given the scarcity of independent external validation cohorts, notable heterogeneity among them, and the observed decline in model performance. Therefore, prospective, multicenter studies following standardized reporting guidelines are a critical future direction. These studies should also focus on developing integrated algorithms that translate histopathological insights into preoperative imaging data to establish robust clinical tools.

## Introduction

Hepatocellular carcinoma (HCC) is the most common pathological subtype of primary liver cancer, accounting for about 90% of cases [1]. Globally, HCC is the fourth leading cause of cancer-related deaths [2]. According to recent epidemiological data, the age-standardized incidence and mortality rates of HCC are highest in Africa and the Western Pacific region. Over 70% of global HCC cases occur in Asia [1]. Despite advancements in treatment modalities for HCC, including liver transplantation, surgical resection, transarterial chemoembolization, local ablation, targeted therapy, and immunotherapy, the 5-year relative survival rate remains below 20% [3]. Even after complete surgical tumor removal, around 50%‐70% of patients with HCC experience tumor recurrence within 5 years postsurgery [4]. Consequently, HCC has become a significant oncological burden that threatens human life.

Microvascular invasion (MVI) is the pathological process by which tumor cells invade the microvascular structures of the liver tissue surrounding an HCC lesion. MVI occurs in approximately 30%‐50% of cases. It is a significant factor in HCC recurrence after surgery and is associated with poor prognoses in patients with HCC [4,5]. Studies have shown that individuals with HCC and MVI-positive status have significantly lower 5-year disease-free survival and overall survival rates than those with MVI-negative status [6, 7]. Notably, MVI status directly influences treatment strategy selection. Wide-range hepatectomy (resection of ≥ 3 liver segments) is recommended for individuals at high preoperative risk for MVI. This method has a significantly lower 5-year cumulative recurrence rate than limited resection (26.6% vs 58.3%, *P*=.040) [8]. However, other studies have found that adjuvant hepatic arterial infusion chemotherapy does not significantly improve the survival of high-risk MVI-positive individuals compared with the untreated group (*P*=.61). Nevertheless, hepatic arterial infusion chemotherapy significantly improves the prognosis of low-risk patients with MVI (*P*<.001) [9]. Furthermore, individuals with MVI undergoing radiofrequency ablation have a significantly higher recurrence risk than those undergoing radical surgery (*P*<.05) [10]. Therefore, accurately identifying MVI preoperatively is significant for formulating individualized, comprehensive treatment regimens and improving patient prognosis [11].

Currently, a definitive diagnosis of MVI relies on a postoperative pathological examination. However, this examination is subject to biases resulting from the quality of slide preparation and interobserver heterogeneity. These factors may lead to diagnostic inaccuracies. Furthermore, the absence of preoperative MVI information restricts its use in personalized treatment decisions. Therefore, developing efficient MVI auxiliary detection tools is crucial for optimizing clinical management of HCC.

While imaging examinations are crucial for evaluating MVI, predictions based on traditional imaging features rely heavily on radiologists’ subjective interpretations. A systematic review and meta-analysis of 19 studies involving 1920 patients revealed that traditional contrast-enhanced features on magnetic resonance imaging (MRI) showed poor overall diagnostic performance in predicting MVI. Only peritumoral enhancement in the arterial phase exhibited moderate diagnostic accuracy. The combined efficacy of other features, such as peritumoral hypointensity in the hepatobiliary phase and irregular margins, was insufficient to meet the requirements for precise preoperative clinical assessment [12]. Recent progress in data mining techniques has accelerated the growth of radiomics. This technique assists in the analysis of imaging features (eg, shape, intensity, and texture) that are difficult for the human eye to perceive. It can overcome some of the limitations of subjectivity. However, radiomic features are mostly low- or mid-level and susceptible to noise interference. They may also not fully reflect tumor heterogeneity [13,14]. Zhang et al [15] noted that radiomics based on single-modality medical imaging is inherently limited. Due to constraints in imaging principles, such approaches can only reflect partial tumor information. Furthermore, when features are extracted using the entire tumor as the region of interest, information about intratumoral heterogeneity is inevitably lost. Additionally, these single-modality radiomic features are susceptible to image noise and variations in scanning parameters, which further compromise the model’s ability to capture tumor heterogeneity comprehensively. In contrast, deep learning (DL) uses multi-layer neural networks and an end-to-end learning mode to directly extract multi-level abstract high-order features from original images. This improves the predictive performance, interpretability, and generalizability of models. DL is expected to provide a new paradigm for the preoperative, noninvasive assessment of MVI [16,17]. However, existing studies often focus on a single imaging modality or have small sample sizes. These studies lack a systematic comparison of DL model performance across different imaging modalities, which limits the interpretation of the advantages of DL in detecting MVI and poses challenges to the development or update of intelligent auxiliary diagnostic tools. Consequently, this meta-analysis was conducted to systematically evaluate the diagnostic efficacy of DL models based on medical images for MVI, as well as to explore the impact of different imaging modalities, validation strategies, and sources of heterogeneity on model performance and generalizability, to provide evidence-based support for the development or update of future intelligent auxiliary diagnostic tools.

## Methods

### Study Registration

This meta-analysis was prospectively registered with the PROSPERO (CRD42024613733). This systematic review and meta-analysis of diagnostic test accuracy was reported in accordance with the PRISMA-DTA (Preferred Reporting Items for Systematic Reviews and Meta-Analyses extension for Diagnostic Test Accuracy studies) [18] guidelines in Checklist 1. Due to the absence of subject information collection and its lack of impact on clinical diagnosis and treatment, ethical approval and informed consent were waived.

### Eligibility Criteria

#### Inclusion Criteria

The inclusion criteria are as follows:

- Original research with full text published in English (including cohort, case-control, and cross-sectional studies).
- MVI status in HCC individuals had to be confirmed by histopathology or biopsy.
- Studies had to develop complete DL models based on medical images to detect MVI status in patients with HCC.
- English-language studies.

#### Exclusion Criteria

The exclusion criteria are as follows:

- Meta-analyses, reviews, guidelines, or expert opinions.
- Only differential factor analysis was implemented without a comprehensive DL model.
- Studies lacking outcome measures of predictive accuracy for the machine learning model, including sensitivity, C-index, accuracy, specificity, precision, *F*_1_-score, and confusion matrix.
- Only image segmentation was performed.

### Search Strategies and Data Sources

We conducted a systematic literature search in accordance with the PRISMA-S (Preferred Reporting Items for Systematic Reviews and Meta-Analyses extension for literature searches; completed checklist is available in Checklist 2) [19]. Relevant English-language publications were retrieved from PubMed, Web of Science, the Cochrane Library, and Embase, with the search covering all records up to October 16, 2025. The search strategy used both MeSH (Medical Subject Headings) and free-text keywords, including MeSH terms such as “Carcinoma, Hepatocellular,” “liver cell carcinoma,” and “deep learning.” Boolean operators were used to integrate MeSH terms and free-text terms, constructing tailored search queries for each database. Furthermore, the reference lists of identified review articles were manually screened to locate any additional eligible studies. No prospective study registries were searched, and no attempts were made to obtain unpublished data or to contact study authors for further information. The complete search strings for each database are provided in Table S1 in Multimedia Appendix 1.

### Study Selection

Retrieved articles were imported into EndNote. After removing duplicates, the remaining articles were reviewed by title and abstract to identify initially eligible articles. Then, the full texts were downloaded and screened to determine the final eligible articles. Two researchers (WF and BQ, with 6 and 4 years of meta-analysis experience, respectively) performed the review independently. Interresearcher agreement during the literature screening was assessed using the κ coefficient (κ=0.93). Any disagreements were resolved in a consensus meeting with a third researcher (SH, with 10 years of experience in meta-analysis).

### Data Extraction

Before data extraction, a standardized spreadsheet was generated. The content to be extracted included the following: publication year, patient source, author, image source, manual segmentation, number of patients with MVI, total number of patients with HCC, number of patients with MVI in the training set, number of patients with HCC in the training set, validation set generation method, number of patients with MVI in the validation set, number of patients with HCC in the validation set, confusion matrix, sensitivity, specificity, precision, and accuracy.

Two researchers (WF and BQ, with 14 and 16 years of medical experience, respectively) carried out the data extraction independently. They then cross-checked their results. Any discrepancies were resolved through consultation with a third researcher (SH, with 13 years of medical experience).

### Risk of Bias in Studies

The Quality Assessment of Diagnostic Accuracy Studies 2 (QUADAS-2) tool was used to evaluate the overall risk of bias (ROB) and applicability of the eligible studies. The QUADAS-2 instrument encompasses 4 domains: index test, patient selection, flow and timing, and reference standard. Each domain includes specific questions. Answers to these questions are categorized as “yes,” “no,” or “unclear.” These answers correspond to ROB ratings of “unclear,” “high,” or “low.” If all key questions within a domain received a “yes” answer, the domain was rated as having a low ROB. If any key question received a “no” answer, a potential ROB was indicated, and the researcher judged the ROB according to established guidelines. An “unclear” rating was assigned when the literature did not provide sufficient detail for the researcher to make a judgment.

Two researchers completed the QUADAS-2 assessment independently. Any discrepancies were resolved through discussion with a third researcher.

### Synthesis Methods

A meta-analysis was performed using a bivariate mixed-effects model based on diagnostic 2×2 tables [20]. For studies that did not report these tables directly, we derived them from the available specificity, sensitivity, positive predictive value, accuracy, *F*_1_-score, and case numbers. Throughout the analysis, the explicitly defined independent validation cohort from each study served as the unit of analysis. Each data point corresponded to distinct and nonoverlapping patient samples, which ensured the independence of the pooled results and mitigated potential bias from data reuse. Using the bivariate mixed-effects model, we computed the pooled estimates for specificity, sensitivity, negative likelihood ratio (LR–), positive likelihood ratio (LR+), diagnostic odds ratio (DOR), and area under the summary receiver operating characteristic (SROC) curve, along with their 95% CIs [20]. The Spearman correlation coefficient was used to evaluate the threshold effect and its contribution to between-study heterogeneity. Small-study effects were assessed using Deeks funnel plot asymmetry test. For subgroups with fewer than 10 studies, a Doi plot was used to informally assess publication bias. The degree of bias was determined based on the absolute value of the Luis Furuya-Kanamori (LFK) index. A value less than 1 suggests minor publication bias, a value between 1 and 2 indicates moderate publication bias, and a value exceeding 2 suggests substantial publication bias. During the meta-analysis, the validation set was used, and subgroup analyses were performed according to the validation set generation method and image type. A *P* value less than .05 was considered statistically significant.

## Results

### Study Selection

**Figure 1.**
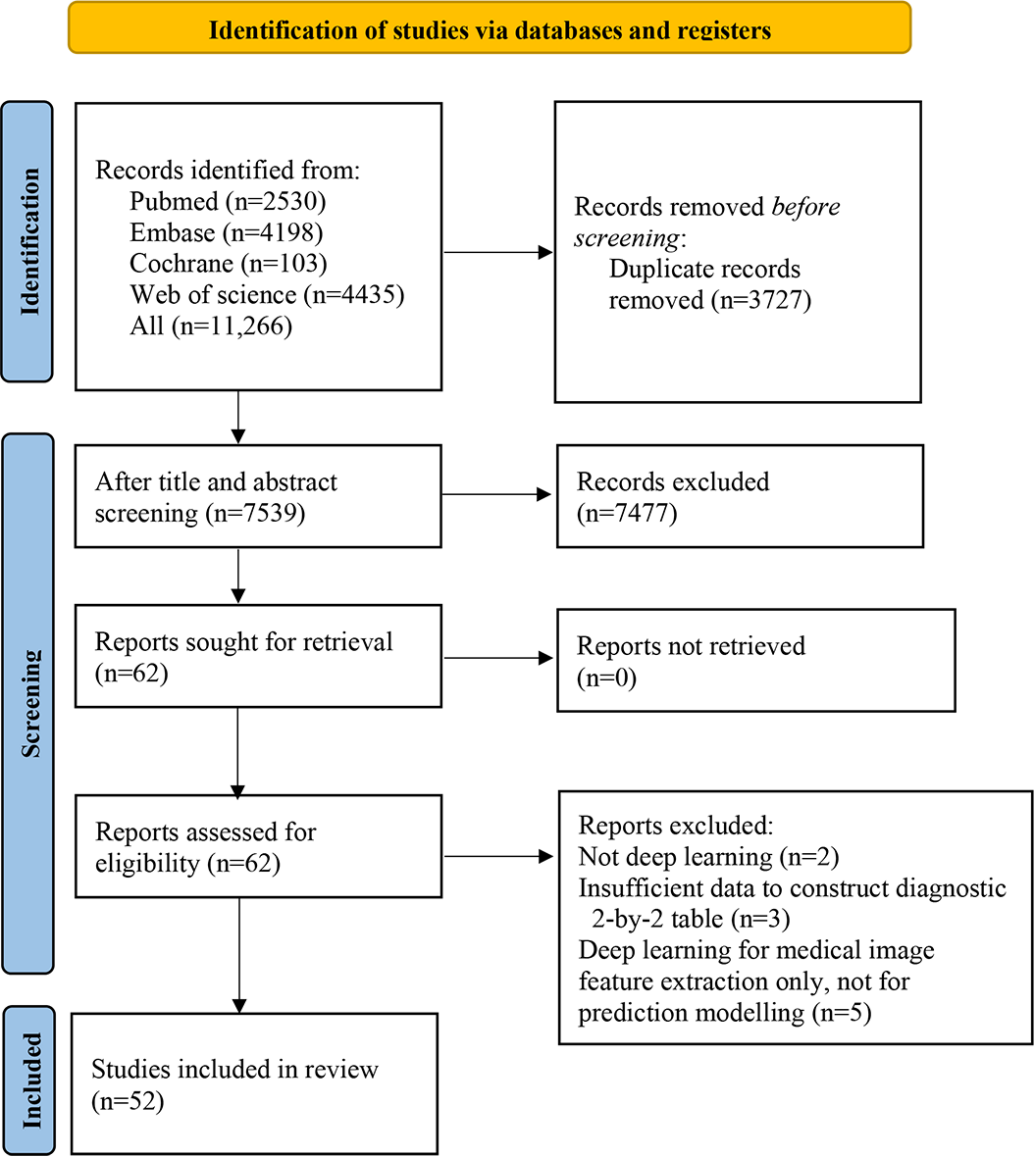
PRISMA (Preferred Reporting Items for Systematic Reviews and Meta-Analyses) flow diagram of the study screening and selection process for this meta-analysis.

Database retrieval yielded 11,266 articles, of which 7539 remained after duplicate removal. Following title and abstract screening, 62 articles were selected for a full-text review. The full-text review subsequently excluded 10 records: 2 non-DL studies, 3 studies with insufficient data to construct diagnostic 2×2 tables, and 5 studies that used DL solely for medical image segmentation without establishing HCC MVI prediction models. Finally, 52 articles [14,16,21-70] met the eligibility criteria. The detailed process is illustrated in Figure 1.

### Study Characteristics

This meta-analysis included 52 studies published between 2019 and 2025. All of the studies used histopathological diagnosis as the gold standard for MVI. All 52 studies were case-control studies, encompassing 19,531 individuals with HCC, of whom 8161 were MVI cases. Regarding population source, 32 (61.5%) of the studies were single-center, 19 (36.5%) studies were multicenter, and 1 (1.9%) study was based on a registry database.

These studies primarily used single-modality imaging techniques for image modeling methods: contrast-enhanced computed tomography (CECT, n=16), contrast-enhanced magnetic resonance imaging (CEMRI, n=19), MRI (n=5), contrast-enhanced ultrasound (CEUS, n=5), and pathological sections (n=2). Five additional studies integrated multimodal imaging (computed tomography + positron emission tomography: 1, CECT +CEMRI: 4) for modeling. Regarding image segmentation methods, 38 of the 52 (73.1%) studies used manual segmentation, while the remaining studies used fully automatic segmentation (n=5) or semiautomatic segmentation (n=9).

Concerning model validation strategies, 23 studies used randomly sampled internal validation, 15 used performance through cross-validation, and 14 used external validation with independent cohorts (Table 1).

**Table 1.** Basic characteristics of the 52 eligible studies evaluating medical image-based deep learning models for preoperatively detecting microvascular invasion (MVI) in patients with hepatocellular carcinoma.

Author (year of publication)	Basic characteristics						Training versus validation cohort characteristics				
	Source of the patients^a^	Imaging modality	Segmentation	Diagnostic criteria for MVI	Patients with MVI	Sample size	Patients with MVI in the training set	Sample size in the training set	Type of validation	Patients with MVI in the validation set	Sample size in the validation set
Zhang et al (2024)^a^ [21]	Single center +registration database (TCGA)^b^	Pathological section	Semiautomatic segmentation	Pathological diagnosis	576	1111	328	530	Random sampling (7:3), external validation (registration database TCGA)	Validation set 1: 143; Validation set 2: 105	Validation set 1: 223; Validation set 2: 358
Lei et al (2024) [22]	Multicenter	CECT^c^+CEMRI^d^	Semiautomatic segmentation	Pathological diagnosis	104	345	89	301	Random sampling (6:4)	15	44
Liu et al (2024) [23]	Single center	CEMRI	Manual segmentation	Pathological diagnosis	123	265	97	211	Random sampling (8:2)	26	54
Zhou et al (2024) [24]	Multicenter	CECT	Semiautomatic segmentation	Pathological diagnosis	54	140	38	98	Random sampling (7:3)	16	42
He et al (2024) [25]	Multicenter	CECT	Semiautomatic segmentation	Pathological diagnosis	299	640	172	368	Internal validation	Validation set 1: 63; Validation set 2: 64	Validation set 1: 134; Validation set 2: 138
Zhong et al (2024) [26]	Single center	CEMRI	Manual segmentation	Pathological diagnosis	82	173	57	120	Random sampling (7:3)	25	53
Wang et al (2024)^a^ [27]	Multicenter	CEMRI	Manual segmentation	Pathological diagnosis	274	725	109	234	Random sampling (4:1); External validation (multicenter)	Validation set 1: 20; Validation set 2.1: 82; Validation set 2.2: 37; Validation set 2.3: 26	Validation set 1: 58; Validation set 2.1: 212; Validation set 2.2: 111; Validation set 2.3: 110
Yu et al (2024)^a^ [28]	Multicenter	CECT	Semiautomatic segmentation	Pathological diagnosis	78	205	39	119	Random sampling (7:3); External validation (multicenter)	Validation set 1: 15; Validation set 2: 24	Validation set 1: 44; Validation set 2: 42
Ma et al (2025) [29]	Single center	CEMRI	Manual segmentation	Pathological diagnosis	52	117	42	94	Random sampling (8:2); 10-fold cross-validation	10	23
Zhang et al (2024)^a^ [30]	Multicenter	CEUS^e^	Manual segmentation	Pathological diagnosis	219	576	175	461	Single center random sampling (8:2); External validation (multicenter)	—^f^	—
Wang et al (2023)^a^ [31]	Multicenter	CECT+CEMRI	Manual segmentation	Pathological diagnosis	150	397	119	297	External validation (multicenter)	31	100
You et al (2023) [32]	Single center	CEMRI	Semiautomatic segmentation	Pathological diagnosis	70	210	56	168	Random sampling (4:1); 5-fold cross-validation	14	42
Qin et al (2023) [33]	Multicenter	CEUS	Manual segmentation	Pathological diagnosis	92	252	71	198	Random sampling (8:2)	21	54
Li et al (2023) [34]	Single center	CEMRI	Manual segmentation	Pathological diagnosis	146	283	117	226	Random sampling (4:1); 5-fold cross-validation	29	57
Cao et al (2023) [35]	Single center	CECT	Manual segmentation	Pathological diagnosis	149	559	120	448	Random sampling (4:1)	29	111
Wang et al (2023) [36]	Single center	CEMRI	Manual segmentation	Pathological diagnosis	109	233	76	163	Random sampling (7:3)	33	70
Ye et al (2023) [37]	Multicenter	CECT+PET^g^	Manual segmentation	Pathological diagnosis	41	100	29	70	Random sampling (7:3); 5-fold cross-validation	12	30
Xu et al (2023) [38]	Single center	CECT	Manual segmentation	Pathological diagnosis	99	305	79	244	Random sampling (8:2); 5-fold cross-validation	20	61
Deng et al (2022) [39]	Single center	CECT+CEMRI	Manual segmentation	Pathological diagnosis	44	103	35	82	Random sampling (4:1); 5-fold cross-validation	9	21
Li et al (2022) [40]	Multicenter	CECT	Manual segmentation	Pathological diagnosis	433	1116	346	892	Random sampling (4:1)	87	224
Chen et al (2022)^a^ [41]	Multicenter	Pathological section	Automatic segmentation	Pathological diagnosis	224	470	137	270	Random sampling external validation (multicenter)	Validation set 1: 43; Validation set 2: 44	Validation set 1: 80; Validation set 2: 120
Zhang et al (2022) [42]	Single center	CEUS	Manual segmentation	Pathological diagnosis	150	436	103	301	Random sampling (3:1); Internal validation	Validation set 1: 35; Validation set 2: 12	Validation set 1: 02; Validation set 2: 33
Liu et al (2022) [43]	Single center	MRI^h^	Manual segmentation	Pathological diagnosis	43	114	28	74	Random sampling	15	40
Sun et al (2022) [44]	Single center	CECT	Semiautomatic segmentation	Pathological diagnosis	134	358	77	193	Random sampling	Validation set 1: 23; Validation set 2: 34	Validation set 1: 61; Validation set 2: 104
Wang et al (2022) [45]	Single center	CECT	Automatic segmentation	Pathological diagnosis	68	138	54	110	Random sampling (8:1:1); 5-fold cross-validation	Validation set 1: 7; Validation set 2: 7	Validation set 1: 14; Validation set 2: 14
Xiao et al (2022)^a^ [46]	Multicenter	CECT	Automatic segmentation	Pathological diagnosis	1103	2096	458	876	Random sampling (3:1); External validation (multicenter)	Validation set 1: 152; Validation set 2: 327; Validation set 3: 166	Validation set 1: 292; Validation set 2: 578; Validation set 3: 350
Yang et al (2022) [47]	Single center	CECT	Manual segmentation	Pathological diagnosis	36	283	25	198	Random sampling (198:85)	11	85
Sun et al (2022) [48]	Single center	CEMRI	Manual segmentation	Pathological diagnosis	185	321	86	149	Internal validation	99	172
Dai et al (2022) [49]	Single center	CECT	Manual segmentation	Pathological diagnosis	215	400	172	320	Random sampling (80:10:10)	Validation set 1: 21; Validation set 2: 22	Validation set 1: 40; Validation set 2: 40
Zhang et al (2021) [50]	Single center	CEMRI	Manual segmentation	Pathological diagnosis	92	237	61	158	Random sampling	31	79
Liu et al (2021)^a^ [51]	Multicenter	CECT	Manual segmentation	Pathological diagnosis	135	473	68	216	Random sampling (70:30); External validation (multicenter)	Validation set 1: 28; validation set 2: 39	Validation set 1: 93; Validation set 2: 164
Wei et al (2021)^a^ [52]	Multicenter	CECT + CEMRI	Manual segmentation	Pathological diagnosis	270	750	216	635	External validation (prospective, multicenter)	54	115
Zhou et al (2021) [53]	Single center	CEMRI	Manual segmentation	Pathological diagnosis	—	114	—	—	Random sampling	—	—
Wang et al (2021) [54]	Single center	MRI	Manual segmentation	Pathological diagnosis	43	100	24	60	Random sampling	19	40
Zeng et al (2021) [55]	Single center	MRI	Manual segmentation	Pathological diagnosis	38	98	25	64	Random sampling; 4-fold cross-validation	13	34
Gao et al (2021) [56]	Single center	MRI	Manual segmentation	Pathological diagnosis	74	225	56	168	Random sampling	18	57
Jiang et al (2020) [57]	Single center	CECT	Manual segmentation	Pathological diagnosis	220	405	176	324	Random sampling (8:2)	44	81
Song et al (2021) [58]	Single center	CEMRI	Manual segmentation	Pathological diagnosis	225	601	174	461	Random sampling	51	140
Men et al (2019) [59]	Single center	CEMRI	Manual segmentation	Pathological diagnosis	28	63	21	47	4-fold cross-validation	7	16
Zhou et al (2022) [60]	Single center	CECT	Manual segmentation	Pathological diagnosis	145	466	97	311	3-fold cross-validation	48	155
Chu et al (2022) [61]	Single center	CEMRI	Manual segmentation	Pathological diagnosis	51	133	35	93	Random sampling (7:3)	16	40
Huang et al (2022) [62]	Single center	MRI	Manual segmentation	Pathological diagnosis	43	114	32	86	4-fold cross-validation	11	28
Wang et al (2025) [63]	Single center	CEUS	Semiautomatic segmentation	Pathological diagnosis	142	318	99	222	Random sampling; 5-fold cross-validation	43	96
Cen et al (2025) [64]	Single center	CECT	Semiautomatic segmentation	Pathological diagnosis	68	192	47	134	Random sampling (7:3)	21	58
Huang et al (2025) [65]	Multicenter	CEMRI	Manual segmentation	Pathological diagnosis	124	300	87	210	Random sampling; 5-fold cross-validation	37	90
Miao et al (2025)^a^ [66]	Multicenter	CECT	Manual segmentation	Pathological diagnosis	206	483	136	311	Random sampling (8:2); External validation (multicenter)	Validation set 1: 32; Validation set 2: 38	Validation set 1: 77; Validation set 2: 95
Zhu et al (2025)^a^ [14]	Multicenter	CEMRI	Manual segmentation	Pathological diagnosis	120	304	90	216	External validation (multicenter, retrospective)	30	88
Dong et al (2025)^a^ [67]	Single center	CEMRI	Manual segmentation	Pathological diagnosis	188	519	100	263	Random sampling (4:1); External validation (multicenter)	Validation set 1: 26; Validation set 2: 27; Validation set 3: 35	Validation set 1: 66; Validation set 2: 93; Validation set 3: 97
Zhang et al (2025) [68]	Single center	CEMRI	Automatic segmentation	Pathological diagnosis	142	270	114	216	5-fold cross-validation	28	54
Zheng et al (2025)^a^ [16]	Multicenter	CEMRI	Automatic segmentation	Pathological diagnosis	292	589	154	317	Random sampling (7:3); External validation (multicenter)	Validation set 1: 52; Validation set 2: 86	Validation set 1: 106; Validation set 2: 166
Zhao et al (2025)^a^ [69]	Multicenter	CEMRI	Manual segmentation	Pathological diagnosis	51	145	25	66	10-fold cross-validation; External validation (multicenter)	26	79
Qin et al (2025) [70]	Single center	CEUS	Manual segmentation	Pathological diagnosis	65	164	44	114	Random sampling (7:3); 10-fold cross-validation	21	50

a Studies used external validation with an independent cohort.

b TCGA: The Cancer Genome Atlas.

c CECT: contrast-enhanced computed tomography.

d CEMRI: contrast-enhanced magnetic resonance imaging.

e CEUS: contrast-enhanced ultrasound.

f Not available.

g PET: positron emission tomography.

h MRI: magnetic resonance imaging.

### ROB in Studies

Regarding patient selection, all eligible studies included consecutive or random cases. According to the QUADAS-2 assessment criteria, this study design carries an inherent high ROB in the “Patient Selection” domain. Consequently, all included studies received a “high” rating for ROB in this domain.

Due to the adoption of supervised DL, model training was based on clear pathological outcomes. However, since DL models predict by extracting inherent image features, rather than directly relying on clinical covariates, their training process’s dependence on known outcomes did not result in diagnostic information leakage. This led to a low ROB. Manual segmentation was used in 38 studies, which could have introduced operator subjectivity and led to a high ROB.

Regarding the implementation of the gold standard, all studies used histopathological diagnosis as the gold standard for MVI, ensuring the objectivity and consistency of disease classification. This indicated a low ROB in the implementation of the gold standard.

Regarding the item “the match between the conduct and interpretation of the index test and the review question” in the QUADAS-2 scale, 5 (9.6%) out of the 52 studies did not directly report specificity values. Since specificity is a key indicator for verifying the match between a DL model and a clinical question, the absence of such data may lead to an incomplete assessment of a model’s diagnostic efficacy and weaken the reliability of a study’s conclusions. Therefore, these 5 studies were determined to have a high ROB. The remaining 47 (90.4%) studies fully reported diagnostic performance indicators, ensuring the transparency in test conduct and interpretation, and indicating a low ROB.

**Figure 2.**
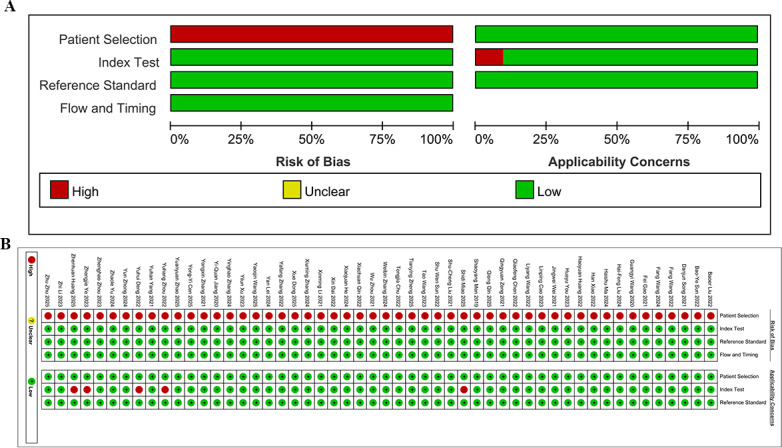
Methodological quality assessment of the included studies based on the Quality Assessment of Diagnostic Accuracy Studies 2 (QUADAS-2) scale. (**A**) Summary, (**B**) Individual studies [14,16,21-70].

There was a reasonable and appropriate time interval between imaging examinations and pathological diagnoses in all eligible studies. Therefore, there did not appear to be a significant impact on the cases’ process (Figure 2).

### Meta-Analysis

#### Overall

**Figure 3.**
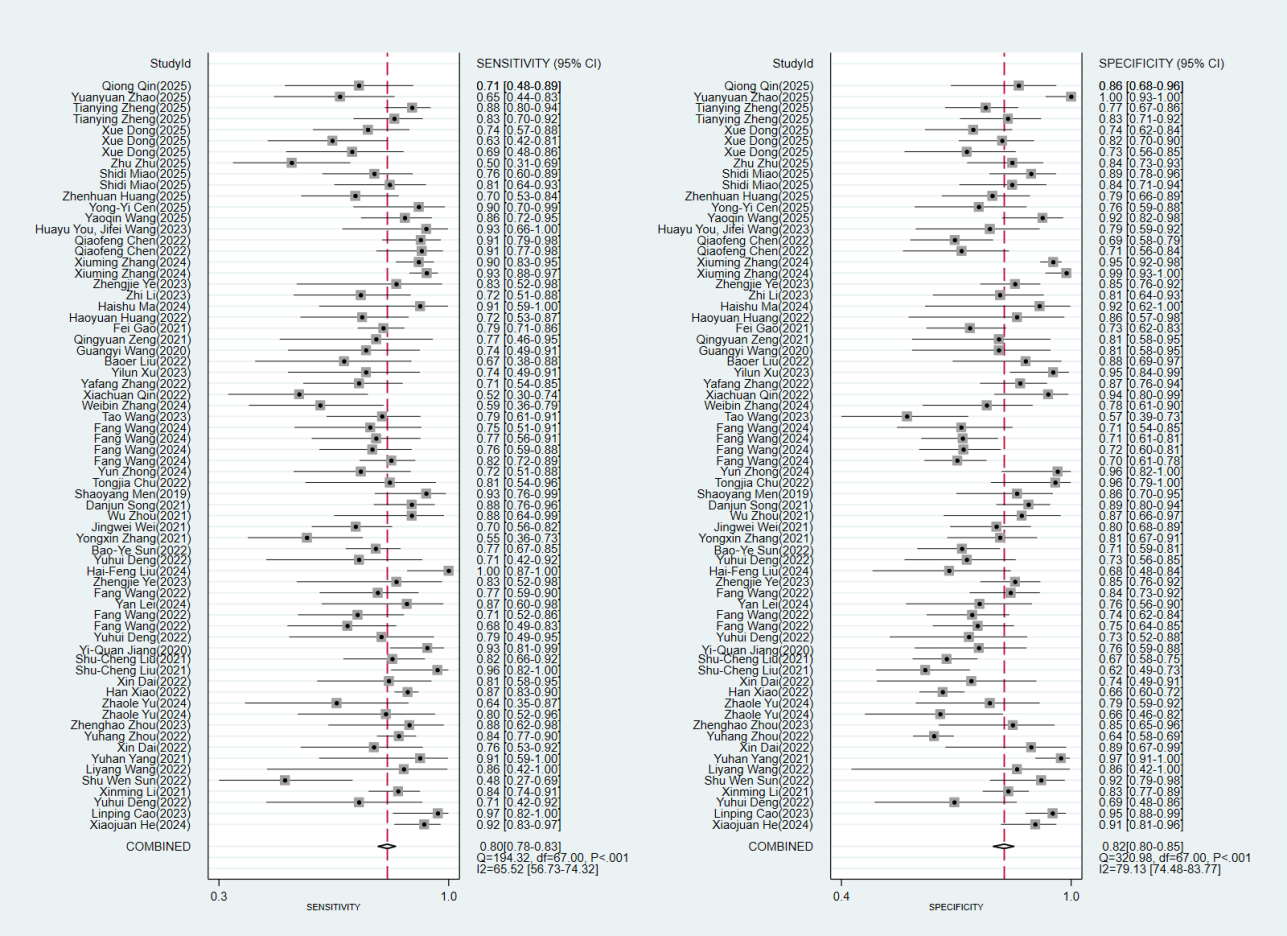
Meta-analysis forest plots: specificity and sensitivity of image-based deep learning in microvascular invasion diagnosis [14,16,21-70].

The model’s accuracy was validated using 68 diagnostic fourfold tables. The Spearman correlation coefficient was 0.02, indicating a minimal threshold effect. This effect accounted for none of the observed between-study heterogeneity. The pooled analysis revealed the following results: sensitivity 0.80 (95% CI 0.78‐0.83, *I*^2^=65.52%), specificity 0.82 (95% CI 0.80‐0.85, *I*^2^=79.13%), LR+4.6 (95% CI 3.9‐5.3), LR- 0.24 (95% CI 0.21‐0.27), DOR 19 (95% CI 15‐25), and SROC 0.88 (95% CI 0.56‐0.98; Table 2 and Figures3  4).

**Table 2.** Meta-analysis results of deep learning for microvascular invasion diagnosis under different image sources and validation set generation methods.

Subgroup	N	SENS^a^ (95% CI)	SPEC^b^ (95% CI)	PLR^c^ (95% CI)	NLR^d^ (95% CI)	DOR^e^ (95% CI)	SROC^f^ (95% CI)	Deeks	Doi
Overall	68	0.80 (0.78-0.83)	0.82 (0.80-0.85)	4.6 (3.9-5.3)	0.24 (0.21-0.27)	19 (15-25)	0.88 (0.56-0.98)	0.77	—^g^
Validation set generation methods									
Internal validation	49	0.82 (0.79-0.85)	0.83 (0.80-0.86)	4.9 (4.1-6.0)	0.22 (0.18-0.26)	23 (17-32)	0.90 (1.00‐0.00)	0.85	—
External validation	19	0.77 (0.72-0.82)	0.80 (0.74-0.85)	3.9 (3.0-5.0)	0.29 (0.23-0.36)	13 (9-20)	0.85 (1.00‐0.00)	0.22	—
The number of cases in the validation set									
<100	38	0.79 (0.75-0.83)	0.83 (0.79-0.87)	4.7 (3.8-5.8)	0.25 (0.20-0.30)	19 (14-26)	0.88 (0.63‐0.97)	0.15	—
≥100	30	0.82 (0.78-0.85)	0.81 (0.77-0.85)	4.3 (3.4-5.5)	0.23 (0.18-0.28)	19 (13-29)	0.88 (0.66‐0.97)	0.5	—
Image sources									
CECT^h^	20	0.84 (0.79-0.88)	0.83 (0.77-0.88)	5.0 (3.6-6.9)	0.19 (0.15-0.25)	26 (16-42)	0.90 (1.00‐0.00)	0.09	
CECT internal validation	16	0.86 (0.80-0.90)	0.85 (0.78-0.90)	5.7 (3.8-8.4)	0.17 (0.12-0.24)	33 (19-59)	0.92 (1.00‐0.00)	0.19	—
CECT external validation	4	0.82 (0.73-0.88)	0.76 (0.64-0.85)	3.4 (2.3-5.0)	0.24 (0.17-0.34)	14 (9-23)	0.86 (1.00‐0.00)	—	−1.14
CEUS^i^	5	0.70 (0.58-0.80)	0.88 (0.82-0.92)	5.6 (3.5-9.0)	0.34 (0.23-0.51)	17 (7-37)	0.89 (1.00‐0.00)	—	0.46
CEMRI^j^	24	0.78 (0.73-0.83)	0.81 (0.76-0.85)	4.0 (3.2-5.0)	0.27 (0.22-0.33)	15 (10-22)	0.86 (1.00‐0.00)	0.15	
CEMRI internal validation	16	0.81 (0.75-0.86)	0.81 (0.75-0.85)	4.3 (3.2-5.6)	0.24 (0.17-0.32)	18 (11-30)	0.88 (1.00‐0.00)	0.17	—
CEMRI external validation	8	0.74 (0.65-0.82)	0.80 (0.71-0.87)	3.7 (2.7-5.2)	0.32 (0.24-0.43)	12 (7-18)	0.84 (1.00‐0.00)	—	1.74
MRI^k^	5	0.76 (0.68-0.82)	0.80 (0.70-0.87)	3.7 (2.5-5.5)	0.30 (0.23-0.40)	12 (7-21)	0.84 (1.00‐0.00)	—	3.11
Multimodal	10	0.74 (0.68-0.80)	0.79 (0.75-0.83)	3.6 (2.9-4.4)	0.33 (0.26-0.41)	11 (7-16)	0.82 (1.00‐0.00)	0.44	
Multimodal internal validation	6	0.79 (0.69-0.87)	0.79 (0.73-0.85)	3.8 (2.8-5.3)	0.26 (0.17-0.41)	15 (7-29)	0.86 (1.00‐0.00)	—	1.6
Multimodal external validation	4	0.71 (0.64-0.78)	0.78 (0.73-0.83)	3.3 (2.6-4.2)	0.36 (0.28-0.47)	9 (6-14)	0.82 (1.00‐0.00)	—	1.93
Pathological Sections	4	0.91 (0.87-0.94)	0.90 (0.68-0.97)	9.2 (2.5-33.6)	0.09 (0.06-0.15)	97 (20-465)	0.92 (1.00‐0.00)	—	3.08

a SENS: sensitivity.

b SPEC: specificity.

c PLR: positive likelihood ratio.

d NLR: negative likelihood ratio.

e DOR: diagnostic odds ratio.

f SROC: summary receiver operating characteristic.

g Not applicable.

h CECT: contrast-enhanced computed tomography.

i CEUS: contrast-enhanced ultrasound.

j CEMRI: contrast-enhanced magnetic resonance imaging.

k MRI: magnetic resonance imaging.

**Figure 4.**
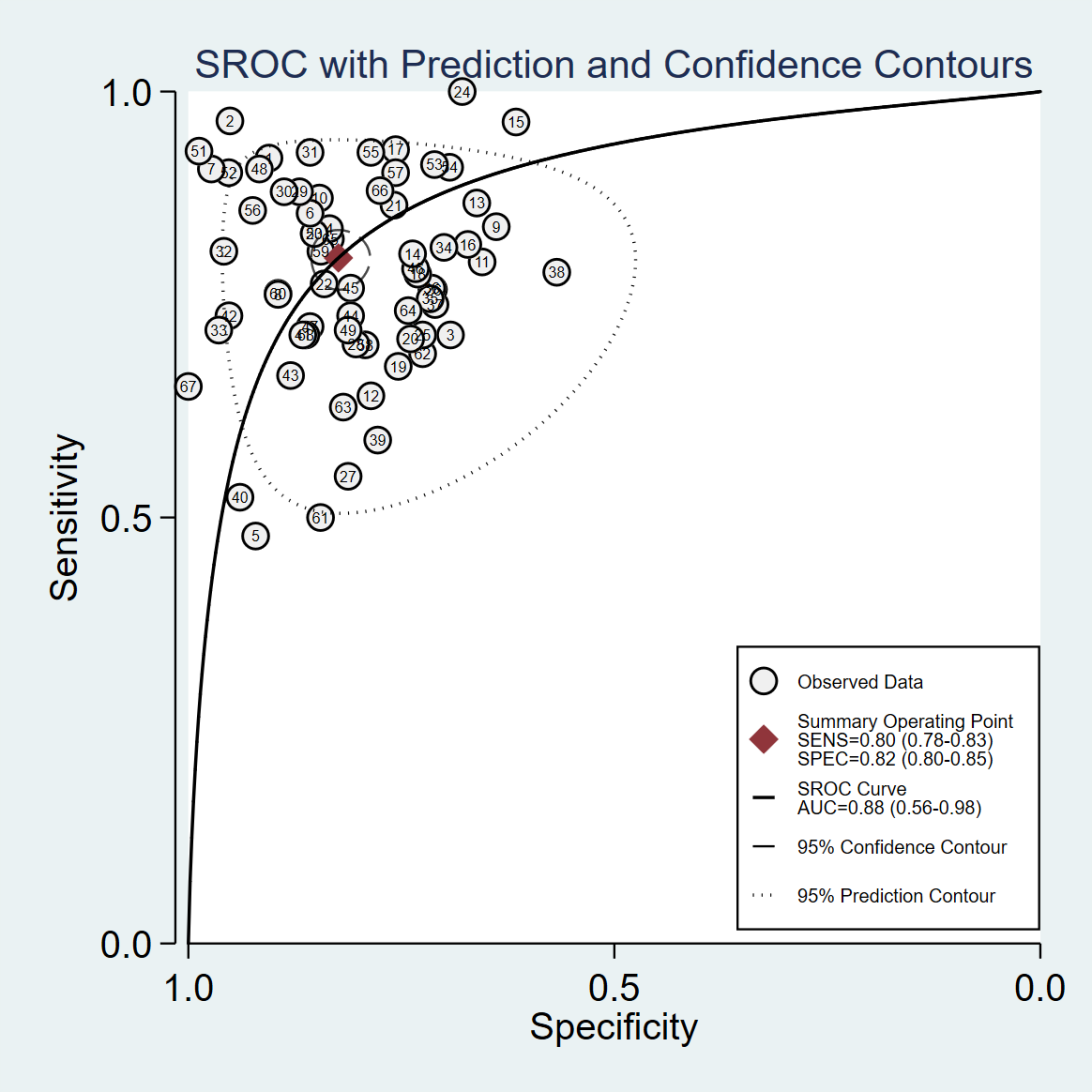
Meta-analysis SROC: specificity and sensitivity of image-based deep learning in microvascular invasion diagnosis. SENS: sensitivity; SPEC: specificity; SROC: summary receiver operating characteristic.

**Figure 5.**
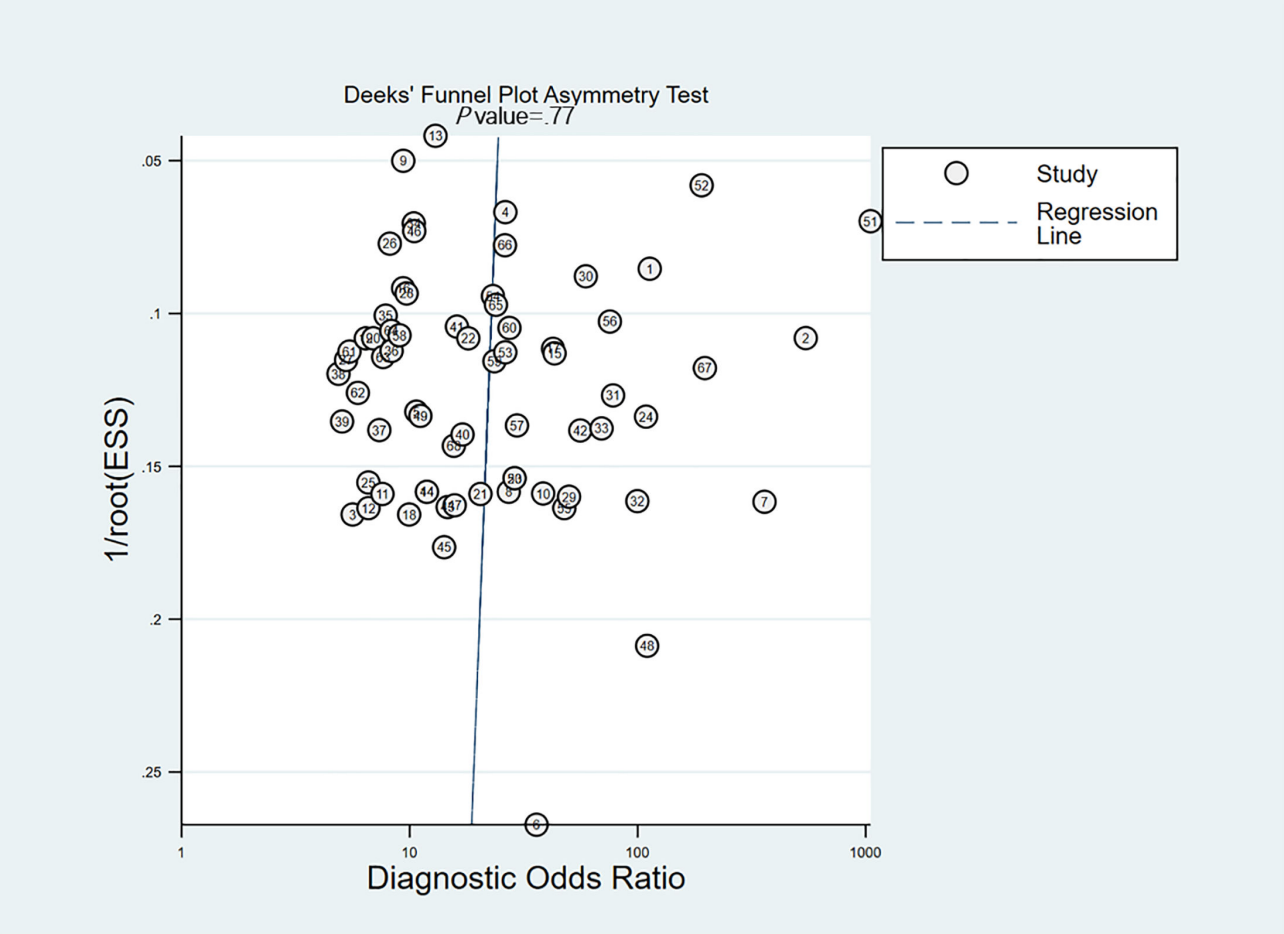
Deeks’ funnel plot from meta-analysis of the specificity and sensitivity of image-based deep learning in microvascular invasion diagnosis.

**Figure 6.**
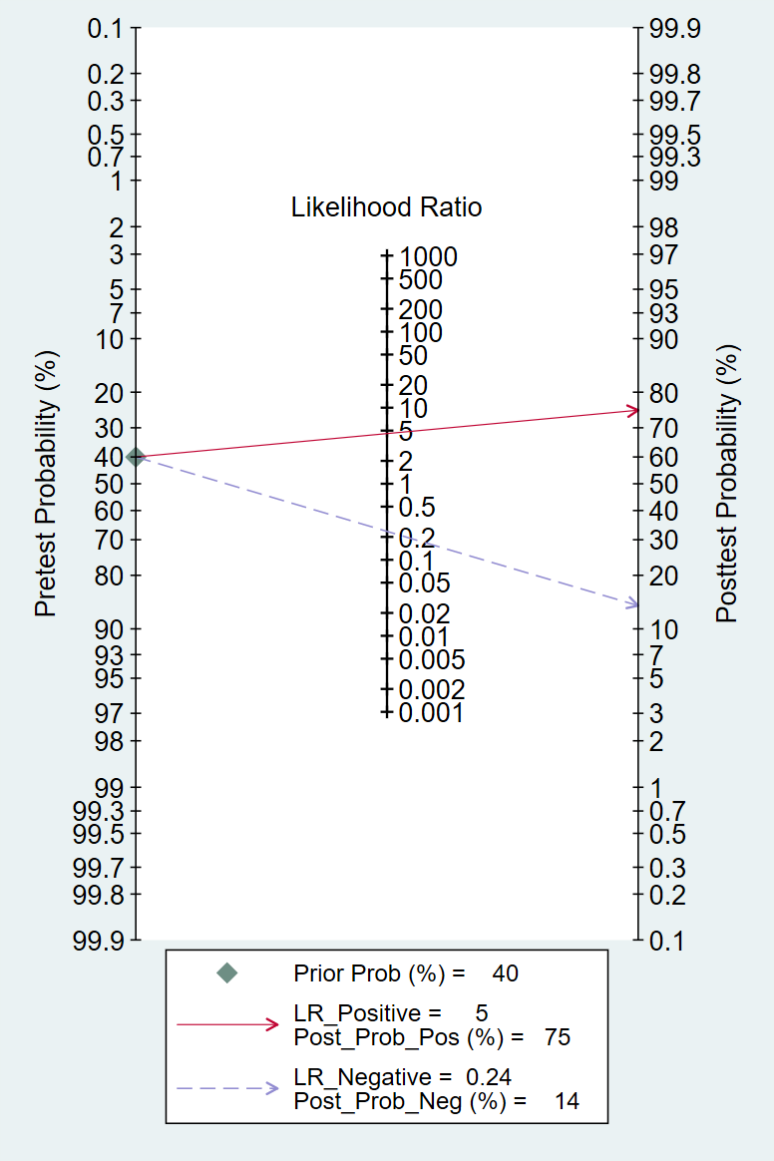
Fagan’s nomogram from meta-analysis of the specificity and sensitivity of image-based deep learning in microvascular invasion diagnosis.

Deeks’ funnel plot indicated no significant small-study effects (*P*=.77, Table 2 and Figure 5). When the prior probability of MVI was set to 40%, the posterior probabilities corresponding to positive and negative DL model detection results were 75% and 14%, respectively. Fagan’s nomogram analysis showed that a positive detection result increased the posterior probability by 35% compared with the prior probability, whereas a negative detection result decreased it by 26% (Figure 6).

#### Subgroup Analysis by Validation Set Generation Method

##### Internal Validation

The model’s accuracy was validated using 49 diagnostic fourfold tables in internal validation. The Spearman correlation coefficient was 0.04, suggesting a minimal threshold effect. This effect accounted for none of the observed between-study heterogeneity. The pooled analysis yielded the following results: sensitivity 0.82 (95% CI 0.79‐0.85, *I*^2^=61.27%), specificity 0.83 (95% CI 0.80‐0.86, *I*^2^=72.22%), LR+4.9 (95% CI 4.1‐6.0), LR–0.22 (95% CI 0.18‐0.26), DOR 23 (95% CI 17‐32), and SROC 0.90 (95% CI 1.00‐0.00; Table 2 and Figures S1A,B in Multimedia Appendix 2).

Deeks’ funnel plot illustrated no significant small-study effects (*P*=.85, Table 2 and Figure S1C in Multimedia Appendix 2). When the prior probability of MVI was set to 40%, the corresponding posterior probabilities for positive and negative DL model detection results were 77% and 13%, respectively. Fagan’s nomogram analysis showed that a positive detection result increased the posterior probability by 37%, compared with the prior probability; a negative detection result decreased the posterior probability by 27% (Figure S1D in Multimedia Appendix 2).

##### External Validation

The model’s accuracy was validated using 19 diagnostic fourfold tables in external validation. The Spearman correlation coefficient was −0.15, indicating a minimal threshold effect. This effect accounted for 2% of the observed between-study heterogeneity. The pooled analysis yielded the following results: sensitivity 0.77 (95% CI 0.72‐0.82, *I*^2^=73.40%), specificity 0.80 (95% CI 0.74‐0.85, *I*^2^=83.19%), LR+3.9 (95% CI 3.0‐5.0), LR–0.29 (95% CI 0.23‐0.36), DOR 13 (95% CI 9‐20), and SROC 0.85 (95% CI 1.00‐0.00; Table 2 and Figures S2A,B in Multimedia Appendix 3).

Deeks’ funnel plot revealed no significant small-study effects (*P*=.22, Table 2 and Figure S2C in Multimedia Appendix 3). When the prior probability of MVI was set to 39%, the corresponding posterior probabilities for positive and negative DL model detection results were 71% and 15%, respectively. Fagan’s nomogram analysis showed that a positive detection result increased the posterior probability by 32% compared with the prior probability, whereas a negative detection result decreased it by 24% (Figure S2D inMultimedia Appendix 3).

##### Validation Set Size < 100 Cases

Among studies with a validation set size <100 cases, the model’s accuracy was validated using 38 diagnostic fourfold tables. The Spearman correlation coefficient was −0.44, suggesting a minimal threshold effect. This effect accounted for 19% of the observed between-study heterogeneity. The pooled analysis yielded the following results: sensitivity 0.79 (95% CI 0.75‐0.83, *I*^2^=56.49%), specificity 0.83 (95% CI 0.79‐0.87, *I*^2^=67.03%), LR+4.7 (95% CI 3.8‐5.8), LR−0.25 (95% CI 0.20‐0.30), DOR 19 (95% CI 14‐26), and SROC 0.88 (95% CI 0.63‐0.97; Table 2 and Figure S3A,B in Multimedia Appendix 4).

Deeks’ funnel plot revealed no significant small-study effects (*P*=.15, Table 2 and Figure S3C in Multimedia Appendix 4). When the prior probability of MVI was set to 41%, the corresponding posterior probabilities for positive and negative DL model detection results were 77% and 15%, respectively. Fagan’s nomogram analysis displayed that a positive detection result increased the posterior probability by 36% compared with the prior probability, whereas a negative detection result decreased it by 26% (Figure S3D in Multimedia Appendix 4).

##### Validation Set Size ≥ 100 Cases

Among studies with a validation set size ≥100 cases, the model’s accuracy was validated using 30 diagnostic fourfold tables. The Spearman correlation coefficient was 0.47, indicating a minimal threshold effect. This effect accounted for 22% of the observed between-study heterogeneity. The pooled analysis yielded the following results: sensitivity 0.82 (95% CI 0.78‐0.85, *I*^2^=69.35%), specificity 0.81 (95% CI 0.77‐0.85, *I*^2^=85.58%), LR+4.3 (95% CI 3.4‐5.5), LR−0.23 (95% CI 0.18‐0.28), DOR 19 (95% CI 13‐29), and SROC 0.88 (95% CI 0.66‐0.97; Table 2 and Figure S4A,B in Multimedia Appendix 5).

Deeks’ funnel plot revealed no significant small-study effects (*P*=.50; Table 2 and Figure S4C in Multimedia Appendix 5). When the prior probability of MVI was set to 39%, the corresponding posterior probabilities for positive and negative DL model detection results were 73% and 13%, respectively. Fagan’s nomogram analysis showed that a positive detection result increased the posterior probability by 34% compared with the prior probability, whereas a negative detection result decreased it by 26% (Figure S4D in Multimedia Appendix 5).

### Subgroup Analysis by Image Source

#### DL Based on CECT

In CECT, the model’s accuracy was validated using 20 diagnostic fourfold tables. The Spearman correlation coefficient was −0.28, suggesting a minimal threshold effect. This effect accounted for 8% of the observed between-study heterogeneity. The pooled analysis revealed the following results: sensitivity 0.84 (95% CI 0.79‐0.88, *I*^2^=71.67%), specificity 0.83 (95% CI 0.77‐0.88, *I*^2^=87.84%), LR+5.0 (95% CI 3.6‐6.9), LR− 0.19 (95% CI 0.15‐0.25), DOR 26 (95% CI 16‐42), and SROC 0.90 (95% CI 1.00‐0.00; Table 2 and Figure S5 in Multimedia Appendix 6). Deeks funnel plot revealed no significant small-study effects (*P*=.09, Table 2).

Among CECT-based models with internal validation, the accuracy was validated using 16 diagnostic fourfold tables. The Spearman correlation coefficient was −0.26, indicating a minimal threshold effect. This effect accounted for 7% of the observed between-study heterogeneity. The pooled analysis yielded the following results: sensitivity 0.86 (95% CI 0.80‐0.90, *I*^2^=71.85%), specificity 0.85 (95% CI 0.78‐0.90, *I*^2^=88.74%), LR+5.7 (95% CI 3.8‐8.4), LR−0.17 (95% CI 0.12‐0.24), DOR 33 (95% CI 19‐59), and SROC 0.92 (95% CI 1.00‐0.00; Table 2 and Multimedia Appendix 7). Deeks’ funnel plot revealed no significant small-study effects (*P*=.19, Table 2).

Among CECT-based models with external validation, the accuracy was validated using four diagnostic fourfold tables. The pooled analysis yielded the following results: sensitivity 0.82 (95% CI 0.73‐0.88, *I*^2^=62.47%), specificity 0.76 (95% CI 0.64‐0.85, *I*^2^=77.88%), LR+3.4 (95% CI 2.3‐5.0), LR− 0.24 (95% CI 0.17‐0.34), DOR 14 (95% CI 9‐23), and SROC 0.86 (95% CI 1.00‐0.00; Table 2 and Multimedia Appendix 7). Subsequent Doi plot analysis showed moderate publication bias among the included studies (LFK index=−1.14, Table 2).

#### DL Based on CEUS

In CEUS, the model’s accuracy was validated using 5 diagnostic fourfold tables. The pooled analysis yielded the following results: sensitivity 0.70 (95% CI 0.58‐0.80, *I*^2^=58.75%), specificity 0.88 (95% CI 0.82‐0.92, *I*^2^=30.18%), LR+5.6 (95% CI 3.5‐9.0), LR−0.34 (95% CI 0.23‐0.51), DOR 17 (95% CI 7‐37), and SROC 0.89 (95% CI 1.00‐0.00; Table 2 and Multimedia Appendix 8). Analysis using the Doi plot revealed minimal publication bias among the included studies (LFK index=0.46, Table 2).

#### DL Based on CEMRI

In CEMRI, the model’s accuracy was validated using 24 diagnostic fourfold tables. The Spearman correlation coefficient was −0.22, suggesting a minimal threshold effect. This effect accounted for 5% of the observed between-study heterogeneity. The pooled analysis yielded the following results: sensitivity 0.78 (95% CI 0.73‐0.83, *I*^2^=61.70%), specificity 0.81 (95% CI 0.76‐0.85, *I*^2^=65.14), LR+4.0 (95% CI 3.2‐5.0), LR− 0.27 (95% CI 0.22‐0.33), DOR 15 (95% CI 10‐22), and SROC 0.86 (95% CI 1.00‐0.00; Table 2 and Multimedia Appendix 9). Deeks funnel plot revealed no significant small-study effects (*P*=0.15, Table 2).

Among CEMRI models with internal validation, the accuracy was validated using 16 diagnostic fourfold tables. The Spearman correlation coefficient was 0.17, indicating a minimal threshold effect. This effect accounted for 3% of the observed between-study heterogeneity. The pooled analysis yielded the following results: sensitivity 0.81 (95% CI 0.75‐0.86, *I*^2^=57.28%), specificity 0.81 (95% CI 0.75‐0.85, *I*^2^=61.32), LR+4.3 (95% CI 3.2‐5.6), LR− 0.24 (95% CI 0.17‐0.32), DOR 18 (95% CI 11‐30), and SROC 0.88 (95% CI 1.00‐0.00; Table 2 and Figure S9 in Multimedia Appendix 10). Deeks funnel plot revealed no significant small-study effects (*P*=.17, Table 2).

Among CEMRI models with external validation, the accuracy was validated using 8 diagnostic fourfold tables. The Spearman correlation coefficient was −0.72, suggesting a significant threshold effect. This effect accounted for 52% of the observed between-study heterogeneity. The pooled analysis yielded the following results: sensitivity 0.74 (95% CI 0.65‐0.82, *I*^2^=70.90%), specificity 0.80 (95% CI 0.71‐0.87, *I*^2^=72.55%), LR+3.7 (95% CI 2.7‐5.2), LR−0.32 (95% CI 0.24‐0.43), DOR 12 (95% CI 7‐18), and SROC 0.84 (95% CI 1.00‐0.00; Table 2 and Figure S9 in Multimedia Appendix 10). Further Doi plot analysis revealed moderate publication bias among the included studies (LFK index=1.74, Table 2).

#### DL Based on MRI

In MRI, the model’s accuracy was validated using 5 diagnostic fourfold tables. The pooled analysis revealed the following results: sensitivity 0.76 (95% CI 0.68‐0.82, *I*^2^=0.00%), specificity 0.80 (95% CI 0.70‐0.87, *I*^2^=0.00%), LR+3.7 (95% CI 2.5‐5.5), LR−0.30 (95% CI 0.23‐0.40), DOR 12 (95% CI 7‐21), and SROC, 0.84 (95% CI 1.00-0.00; Table 2 and Multimedia Appendix 11). Analysis using the Doi plot revealed substantial publication bias among the included studies (LFK index=3.11, Table 2).

#### DL Based on Multimodal Imaging

In multimodal medical images, the model’s accuracy was validated using 10 diagnostic fourfold tables. The pooled analysis yielded the following results: sensitivity 0.74 (95% CI 0.68‐0.80, *I*^2^=0.00%), specificity 0.79 (95% CI 0.75‐0.83, *I*^2^=11.32%), LR+3.6 (95% CI 2.9‐4.4), LR− 0.33 (95% CI 0.26‐0.41), DOR 11 (95% CI 7‐16), and SROC 0.82 (95% CI 1.00‐0.00; Table 2 and Multimedia Appendix 12). Deeks’ funnel plot revealed no significant small-study effects (*P*=.44, Table 2).

In multimodal internal validation, the model’s accuracy was validated using 6 diagnostic fourfold tables. The pooled analysis revealed the following results: sensitivity 0.79 (95% CI 0.69‐0.87, *I*^2^=0.00%), specificity 0.79 (95% CI 0.73‐0.85, *I*^2^=31.69%), LR+3.8 (95% CI 2.8‐5.3), LR− 0.26 (95% CI 0.17‐0.41), DOR 15 (95% CI 7‐29), and SROC 0.86 (95% CI 1.00‐0.00; Table 2 and Multimedia Appendix 13). Analysis using the Doi plot revealed moderate publication bias among the included studies (LFK index=1.6; Table 2).

In multimodal external validation, the model’s accuracy was validated using 4 diagnostic fourfold tables. The pooled analysis showed the following results: sensitivity 0.71 (95% CI 0.64‐0.78, *I*^2^=0.00%), specificity 0.78 (95% CI 0.73‐0.83, *I*^2^=0.00%), LR+3.3 (95% CI 2.6‐4.2), LR− 0.36 (95% CI 0.28‐0.47), DOR 9 (95% CI 6‐14), and SROC 0.82 (95% CI 1.00‐0.00; Table 2 and Multimedia Appendix 13). Subsequent Doi plot analysis indicated moderate publication bias (LFK index=1.93; Table 2).

#### DL Based on Pathological Sections

In pathological sections, the model’s accuracy was validated using 4 diagnostic fourfold tables. The pooled analysis revealed the following results: sensitivity 0.91 (95% CI 0.87‐0.94, *I*^2^=0.00%), specificity 0.90 (95% CI 0.68‐0.97, *I*^2^=94.83%), LR+9.2 (95% CI 2.5‐33.6), LR− 0.09 (95% CI 0.06‐0.15), DOR 97 (95% CI 20‐465), and SROC 0.92 (95% CI 1.00‐0.00; Table 2 and Multimedia Appendix 14). Further Doi plot analysis revealed substantial publication bias (LFK index=3.08; Table 2).

## Discussion

### Summary of the Main Findings

The current meta-analysis revealed that the modeling methods used for DL-based prediction of HCC MVI primarily used CECT, CEUS, CEMRI, MRI, multimodal imaging, and pathological image techniques. DL models based on medical imaging showed favorable overall diagnostic performance in predicting HCC MVI, with a pooled sensitivity of 0.80 (95% CI 0.78‐0.83) and specificity of 0.82 (95% CI 0.80‐0.85). Further analysis across imaging modalities revealed that CECT-based models achieved the highest diagnostic efficacy, showing a sensitivity of 0.84 (95% CI 0.79‐0.88) and specificity of 0.83 (95% CI 0.77‐0.88). Models based on CEUS exhibited particularly high specificity (0.88, 95% CI 0.82‐0.92). Furthermore, models using pathological slides, considered the diagnostic reference standard, attained the highest overall performance, with a sensitivity of 0.91 (95% CI 0.87‐0.94) and specificity of 0.90 (95% CI 0.68‐0.97). Therefore, these models appear promising as a diagnostic approach for MVI in HCC.

### Comparison With Other Previous Reviews

This study also noted that some researchers have discussed the use of machine learning for MVI in HCC. Xiao et al [71] and Liang et al [72], who focused on MRI and ultrasound radiomics, respectively, validated the predictive potential of single modalities, with pooled area under the curves (AUCs) of 0.87 and 0.81, respectively. However, their analyses included a limited number of studies and focused only on a single imaging modality, limiting the generalizability of their conclusions. Li et al [73] integrated multiple imaging modalities across 22 studies (involving 4129 participants), reporting a pooled AUC of 0.90 for radiomic models. However, their analysis primarily incorporated traditional machine learning models and did not stratify performance based on the validation set generation method. This limitation may lead to an overly optimistic assessment of model generalizability.

Unlike previous systematic reviews that focused on traditional machine learning models, our meta-analysis focuses on the value of DL algorithms for diagnosing HCC MVI. Methodologically, this study provides a deeper exploration of the sources of performance heterogeneity through subgroup analyses based on the validation set generation method and image source. A particular methodological strength is the inclusion of pathological sections, the diagnostic gold standard, as a benchmark for performance optimization. This meta-analysis synthesized data from 52 studies involving 19,531 patients and provided more robust and reliable conclusions than those from analyses with smaller sample sizes.

### Influence of Imaging Modalities on DL

The imaging modalities used to construct DL models can be categorized into 2 main types: noninvasive and invasive. This study showed that the properties of different modalities and their ability to extract biological features directly affected the diagnostic efficacy of corresponding models.

Among noninvasive imaging modalities, CECT can effectively capture tumor heterogeneity, enhancement patterns, and peritumoral microenvironment changes due to its high spatial resolution and multi-phase dynamic imaging capabilities. These features support the superior diagnostic performance of DL models (AUC=0.90) [74,75]. Models developed from CEUS achieved commendable performance (AUC=0.89) and exhibited a high pooled specificity (0.88). This finding suggests that CEUS’s real-time hemodynamic properties may be valuable in ruling out MVI-negative cases. However, its comparatively lower sensitivity (0.70) concurrently suggests ongoing challenges in consistently identifying MVI-positive features. Integrating Sonazoid-based functional imaging with conventional ultrasound characteristics and serum markers in the future may be a promising way to improve performance [76]. CEMRI has unique advantages in depicting tumor boundaries and detecting subtle changes in the peritumoral liver parenchyma due to its excellent soft tissue contrast [77]. The model’s performance (sensitivity: 0.78, specificity: 0.81, AUC=0.86) surpasses the assessment that relies on traditional visual features, as reported by Wu et al [78] (sensitivity: 0.55, specificity: 0.87, AUC=0.80). However, its widespread adoption is limited by long examination times, high costs, and dependence on equipment. Nonenhanced MRI models also demonstrate predictive potential (sensitivity: 0.76, specificity: 0.80, AUC=0.84). Notably, multimodal fusion models, which are designed to integrate complementary information, have not demonstrated significant advantages in the studies included (AUC=0.82). This may be due to the simplified fusion strategies or data heterogeneity.

Among invasive imaging modalities, DL models based on pathological sections demonstrated the highest diagnostic efficacy in this study (AUC=0.92). This highlights the gold-standard status of pathology in evaluating MVI. However, the inherent invasiveness of pathological examination precludes its use in preoperative decision-making. Therefore, a central challenge for future research is to effectively transfer and integrate the gold-standard-level diagnostic insights from pathological sections into preoperative, noninvasive imaging. This objective requires leveraging advanced methodologies, such as transfer learning, cross-modal fusion, and generative models. Augmenting existing advantageous modalities, such as CECT, with these advancements could ultimately pave the way for a clinically viable preoperative “virtual biopsy.”

### Image Segmentation

Accurate image segmentation is essential for building reliable DL models. However, the literature incorporated in this meta-analysis primarily relies on manual segmentation. This method can introduce subjective bias (38/52, 73.1%). While advanced network architectures have achieved expert-level precision in segmenting normal liver tissue, as demonstrated by Dice coefficients ranging from 0.968 to 0.982 [79,80], comparative data from the Liver Tumor Segmentation challenge reveal significant discrepancies. Specifically, the accuracy of liver tumor segmentation (Dice: 0.739) is considerably lower than that of liver parenchyma segmentation (Dice: 0.963) [81]. This discrepancy primarily stems from the heterogeneity of HCC lesions, suboptimal image contrast, and a lack of high-quality annotated data. Concurrently, selecting the appropriate segmentation strategy is critical. While 3D segmentation comprehensively captures spatial heterogeneity, the more clinically feasible 2D approach sacrifices substantial volumetric information [82]. Given the demand for submillimeter-level precision in MVI prediction, there are 2 key technological pathways for enhancing model stability. First, exploring segmentation paradigms that require fewer annotations, such as weakly or self-supervised learning, can reduce dependency on annotations. Second, developing novel network architectures designed specifically to address HCC heterogeneity and boundary ambiguity is equally crucial.

### Validation Set Generation Method

The rigor of validation strategies is paramount for evaluating the real-world generalizability of DL models in predicting MVI of HCC [83,84]. The present analysis reveals that, despite exemplary performance during internal validation, consistent performance declines emerge in independent external validation cohorts. This finding clearly shows that relying too much on internal validation can lead to overestimating a model’s true efficacy [85]. Concern regarding validation strategies is not unique to DL research. A recent meta-analysis [86] that focused on MRI-based radiomics for predicting HCC recurrence and MVI similarly concluded that the current predominance of internal validation results in an overestimation of model generalizability as well. Consequently, any proclaimed superior performance may substantially diminish when confronting real-world heterogeneity, if the evaluation framework remains confined to internal validation, irrespective of the underlying algorithm—be it DL or radiomics. Therefore, promoting rigorous external validation and establishing standardized, cross-institutional imaging protocols are essential steps toward reliable clinical translation in this field.

### Heterogeneity Analysis

There is substantial heterogeneity among the included studies. Notably, high levels of heterogeneity persist within subgroups, even after stratifying analyses by imaging modality and validation strategy. This observation objectively reflects the inherent complexity of artificial intelligence (AI)–based medical imaging research and is a common challenge for meta-analyses in this field. The heterogeneity primarily stems from 3 levels. Technically, variations in critical parameters, including imaging equipment, magnetic field strength, and slice thickness, directly influence image texture and quality. These variations are a significant technical source of variability in model performance. Methodologically, diversity in study design, validation strategies, segmentation techniques, and DL network architectures introduces additional variation in model construction and performance interpretation. Clinically, differences in patient populations regarding geographic distribution, underlying liver disease etiology, and disease stage may also affect model performance and generalizability. While this heterogeneity limits the direct interpretability of the pooled results to some extent, it accurately reflects the diversity in methodology and clinical practice within the field. Future investigations should adhere to the Findable, Accessible, Interoperable, and Reusable principles, providing detailed reporting of imaging acquisition parameters, model architectures, and training specifics. Such comprehensive reporting will facilitate the in-depth exploration of heterogeneity sources via methods such as meta-regression. This will promote the identification of key influencing factors and the standardization of methodologies.

### Methods for ROB Assessment

This systematic review used a composite strategy to assess the ROB. First, all included studies were rigorously evaluated according to the QUADAS-2 guidelines. The results showed that all primary studies were rated as having a high ROB in the “Patient Selection” domain of QUADAS-2 due to the widespread use of the retrospective case-control design. While this outcome aligns with the QUADAS-2 assessment principles, it also reveals a limitation of the tool when evaluating machine learning-based diagnostic studies that use retrospective data. The tool struggles to differentiate nuances in data construction quality among studies.

To conduct a more granular assessment of data-level bias risk, a supplemental analysis was performed using items from the Quality Assessment of Diagnostic Accuracy Studies for Artificial Intelligence (QUADAS-AI) tool targeting “Study Participant Selection.” The QUADAS-AI tool provides more detailed criteria for this dimension, including an explicit description of data source, size, and quality characteristics, use of open-source datasets, a clear rationale for splitting data into training, validation, and test sets, performance of image preprocessing, and provision of scanner model information. The analysis using QUADAS-AI items showed that all 52 studies appropriately described the source, size, and quality of the input data and clearly defined the patient inclusion criteria. Among these studies, only one used an open-source dataset. All studies provided a rationale for the data split. Image preprocessing was performed in all studies. However, 11 studies did not report the scanner model used for image acquisition (Table S2 in Multimedia Appendix 15 [14,16,21-70]).

### Advantages and Limitations

A primary strength of this research is that it is the first large-scale, systematic meta-analysis to evaluate medical imaging-based DL models for predicting MVI in HCC. The analysis included 52 studies with 19,531 patients, providing the field with comprehensive evidence. The analytical process strictly adhered to the PRISMA guidelines, and bias risk was evaluated using QUADAS-2. These measures ensured methodological rigor and transparency. However, several limitations warrant consideration. First**,** the training cohorts in most primary studies were small. Only 3 studies had a sample size greater than 1000 patients. The robustness of DL models depends heavily on large volumes of high-quality data. Therefore, restricted training sample sizes are a potential methodological limitation. This likely contributes to overfitting in some models, which is probably an internal reason for the observed performance degradation during external validation. This finding underscores the fundamental importance of acquiring large-scale, high-quality datasets to enhance model generalizability in the development of current DL models. Second, the 95% CIs for the pooled AUCs and for most subgroup analyses were exceptionally wide. This constrained the interpretation of the result precision to some extent. This primarily stems from the significant heterogeneity among the included studies. The limited number of studies in subgroup analyses exacerbated data sparsity. Third, Doi plot analyses for some subgroups indicated moderate to substantial publication bias. These analyses showed that the pooled results for these subgroups may be influenced by unpublished negative studies, which poses a risk of overestimating diagnostic performance. Fourth, the rigor of the validation strategies needs to be improved. Most studies relied on internal validation. Only a few conducted stringent external validation. This reliance may introduce an optimistic bias into the overall assessment of the models’ real-world generalizability. While the overall performance on external validation sets was discussed, the limited quantity of external validation data prevented a more in-depth subgroup analysis of validation strategies by different imaging modalities. Fifth, the vast majority of primary studies inadequately reported model calibration metrics or details about network complexity. This omission hinders a quantitative, systematic evaluation of predictive reliability and overfitting risk at the review level and impacts the comprehensive assessment of clinical applicability. This reflects a general deficiency in the transparency of methodological reporting within the current research landscape.

### Clinical Implications and Future Perspectives

This study indicates that medical imaging-based DL models, particularly those using preoperative CECT, demonstrate promising diagnostic performance in predicting MVI of HCC. These models have the potential to assist in personalized surgical planning. However, translating them into clinical practice faces multiple challenges. One primary issue is establishing clinical decision thresholds. While this analysis quantified predictive probabilities via Fagan nomograms, there is currently a lack of evidence-based guidelines defining “at what predicted probability of MVI the surgical margin should be adjusted.” Future work must integrate clinical outcome data and empirically explore the net benefit of different thresholds using methods such as decision curve analysis. Second, these models’ generalizability and deployment feasibility need urgent enhancement. Performance degradation during external validation suggests susceptibility to variations in imaging protocols, equipment, and patient populations. Furthermore, model interpretability is crucial for gaining clinical trust. This necessitates developing transparent methods for presenting decision rationale.

To bridge the gap between “high performance” and “high utility,” future efforts must focus on 3 interconnected levels. At the research level, the focus must shift from model construction to rigorous, prospective, multicenter validation to unequivocally assess generalizability. At the algorithmic level, it is essential to explore cross-modal information fusion, especially using transfer learning, to bring “gold standard”–level diagnostic insights from histopathological sections to preoperative, noninvasive imaging. At the clinical level, establishing standardized, cross-institutional imaging protocols and developing decision support systems that integrate seamlessly into clinical workflows is imperative. This integrated approach is vital for reliably translating technology from innovation to tangible patient benefit.

### Conclusions

This systematic review and meta-analysis demonstrate that medical imaging-based DL models, especially those leveraging preoperative CECT, hold significant promise for the noninvasive preoperative prediction of MVI in HCC. Unlike previous reviews that focused on radiomics or single imaging modalities, this study conducted a comprehensive comparison across multiple modalities. The study also emphasizes the critical role of external validation in the real-world generalizability of a model. However, substantial heterogeneity across studies and the performance degradation observed during independent external validation suggest that their generalizability to the real world must be confirmed through more rigorous study designs. Consequently, future research should prioritize establishing model robustness via prospective, multicenter external validation, coupled with efforts to standardize methodologies and improve reporting transparency. A critical step toward reliable clinical translation and achieving the ultimate goal of a “virtual biopsy” is developing algorithms that can translate pathology-grade diagnostic insights into preoperative, noninvasive imaging.

## Supplementary material

10.2196/82000 Multimedia Appendix 1 Flow diagram illustrating the search strategy.

10.2196/82000 Multimedia Appendix 2 (A) Meta-analysis forest plots: specificity and sensitivity of image-based deep learning (DL) in microvascular invasion (MVI) diagnosis in internal validation; (B) Meta-analysis summary receiver operating characteristic: specificity and sensitivity of image-based DL in MVI diagnosis in internal validation; (C) Deeks funnel plot from meta-analysis of specificity and sensitivity of image-based DL in MVI diagnosis in internal validation; (D) Fagan nomogram from meta-analysis of specificity and sensitivity of image-based DL in MVI diagnosis in internal validation.

10.2196/82000 Multimedia Appendix 3 (A) Meta-analysis forest plots: specificity and sensitivity of image-based deep learning (DL) in microvascular invasion (MVI) diagnosis in external validation; (B) Meta-analysis summary receiver operating characteristic: specificity and sensitivity of image-based DL in MVI diagnosis in external validation; (C) Deeks funnel plot from meta-analysis of specificity and sensitivity of image-based DL in MVI diagnosis in external validation; (D) Fagan nomogram from meta-analysis of specificity and sensitivity of image-based DL in MVI diagnosis in external validation.

10.2196/82000 Multimedia Appendix 4 (A) Meta-analysis forest plots: specificity and sensitivity of image-based deep learning (DL) in microvascular invasion (MVI) diagnosis in Validation set size of <100 cases; (B) Meta-analysis summary receiver operating characteristic: specificity and sensitivity of image-based DL in MVI diagnosis in Validation set size < 100 cases; (C) Deeks funnel plot from meta-analysis of specificity and sensitivity of image-based DL in MVI diagnosis in the validation set size of <100 cases; (D) Fagan nomogram from meta-analysis of specificity and sensitivity of image-based DL in MVI diagnosis in the validation set size of <100 cases.

10.2196/82000 Multimedia Appendix 5 (A) Meta-analysis forest plots: specificity and sensitivity of image-based deep learning (DL) in microvascular invasion (MVI) diagnosis in Validation set size of ≥100 cases; (B) Meta-analysis summary receiver operating characteristic: specificity and sensitivity of image-based DL in MVI diagnosis in Validation set size of ≥100 cases; (C) Deeks funnel plot from meta-analysis of specificity and sensitivity of image-based DL in MVI diagnosis in the validation set size ≥100 cases; (D) Fagan nomogram from meta-analysis of specificity and sensitivity of image-based DL in MVI diagnosis in Validation set size of ≥100 cases.

10.2196/82000 Multimedia Appendix 6 (A) Meta-analysis forest plots: specificity and sensitivity of contrast-enhanced computed tomography (CECT)–based deep learning (DL) in microvascular invasion (MVI) diagnosis; (B) Meta-analysis SROC: specificity and sensitivity of CECT-based DL in MVI diagnosis.

10.2196/82000 Multimedia Appendix 7 (A) Meta-analysis forest plots: specificity and sensitivity of contrast-enhanced computed tomography (CECT)–based deep learning (DL) in microvascular invasion (MVI) diagnosis in internal validation; (B) Meta-analysis SROC: specificity and sensitivity of CECT-based DL in MVI diagnosis in internal validation; (C) Meta-analysis forest plots: specificity and sensitivity of CECT-based DL in MVI diagnosis in external validation; (D) Meta-analysis summary receiver operating characteristic: specificity and sensitivity of CECT-based DL in MVI diagnosis in external validation.

10.2196/82000 Multimedia Appendix 8 (A) Meta-analysis forest plots: specificity and sensitivity of contrast-enhanced ultrasound (CEUS)–based deep learning (DL) in microvascular invasion (MVI) diagnosis; (B) Meta-analysis summary receiver operating characteristic: specificity and sensitivity of CEUS-based DL in MVI diagnosis.

10.2196/82000 Multimedia Appendix 9 (A) Meta-analysis forest plots: specificity and sensitivity of contrast-enhanced magnetic resonance imaging (CEMRI)–based deep learning (DL) in microvascular invasion (MVI) diagnosis; (B) Meta-analysis summary receiver operating characteristic: specificity and sensitivity of CEMRI-based DL in MVI diagnosis.

10.2196/82000 Multimedia Appendix 10 (A) Meta-analysis forest plots: specificity and sensitivity of contrast-enhanced magnetic resonance imaging (CEMRI)–based deep learning (DL) in microvascular invasion (MVI) diagnosis in internal validation; (B) Meta-analysis summary receiver operating characteristic: specificity and sensitivity of CEMRI-based DL in MVI diagnosis in internal validation; (C) Meta-analysis forest plots: specificity and sensitivity of CEMRI-based DL in MVI diagnosis in external validation; (D) Meta-analysis SROC: specificity and sensitivity of CEMRI-based DL in MVI diagnosis in external validation.

10.2196/82000 Multimedia Appendix 11 (A) Meta-analysis forest plots: specificity and sensitivity of magnetic resonance imaging (MRI)–based deep learning (DL) in microvascular imaging (MVI) diagnosis; (B) Meta-analysis summary receiver operating characteristic: specificity and sensitivity of MRI-based DL in MVI diagnosis.

10.2196/82000 Multimedia Appendix 12 (A) Meta-analysis forest plots: specificity and sensitivity of multimodal imaging-based deep learning (DL) in microvascular invasion (MVI) diagnosis; (B) Meta-analysis summary receiver operating characteristic: specificity and sensitivity of multimodal imaging-based DL in MVI diagnosis.

10.2196/82000 Multimedia Appendix 13 (A) Meta-analysis forest plots: specificity and sensitivity of multimodal imaging-based deep learning (DL) in microvascular invasion (MVI) diagnosis in internal validation; (B) Meta-analysis summary receiver operating characteristic (SROC): specificity and sensitivity of multimodal imaging-based DL in MVI diagnosis in internal validation; (C) Meta-analysis forest plots: specificity and sensitivity of multimodal imaging-based DL in MVI diagnosis in external validation; (D) Meta-analysis SROC: specificity and sensitivity of multimodal imaging-based DL in MVI diagnosis in external validation.

10.2196/82000 Multimedia Appendix 14 (A) Meta-analysis forest plots: specificity and sensitivity of pathological sections-based deep learning (DL) in microvascular invasion (MVI) diagnosis; (B) Meta-analysis summary receiver operating characteristic: specificity and sensitivity of pathological sections-based DL in MVI diagnosis.

10.2196/82000 Multimedia Appendix 15 Summary of bias risk for each study included in the paper according to the QUADAS-AI (artificial intelligence–specific quality assessment of diagnostic accuracy studies) domains.

10.2196/82000 Checklist 1 PRISMA-DTA checklist.

10.2196/82000 Checklist 2 PRISMA-S checklist.

## Abbreviations

AI: artificial intelligence
AUC: area under the curve
CECT: contrast-enhanced computed tomography
CEMRI: contrast-enhanced magnetic resonance imaging
CEUS: contrast-enhanced ultrasound
DL: deep learning
DOR: diagnostic odds ratio
HCC: hepatocellular carcinoma
LFK: Luis Furuya-Kanamori
LR: likelihood ratio
MeSH: Medical Subject Headings
MRI: magnetic resonance imaging
MVI: microvascular invasion
PRISMA-DTA: Preferred Reporting Items for Systematic Reviews and Meta-Analyses extension for Diagnostic Test Accuracy
PRISMA-S: Preferred Reporting Items for Systematic Reviews and Meta-Analyses extension for literature searches
QUADAS-2: Quality Assessment of Diagnostic Accuracy Studies 2
QUADAS-AI: Quality Assessment of Diagnostic Accuracy Studies for Artificial Intelligence
ROB: risk of bias
SROC: summary receiver operating characteristic
