# Supplementary material for: Accuracy of Medical Image–Based Deep Learning for Detecting Microvascular Invasion in Hepatocellular Carcinoma: Systematic Review and Meta-Analysis
Source: J Med Internet Res. 2026 Mar 2;28:e82000. doi: 10.2196/82000 (PMC12954728; doi:10.2196/82000)
Supplement: Multimedia Appendix 1 [file jmir-v28-e82000-s001.docx]

# Table S1 Literature search strategy

**1.Pubmed**

| Search number | Query |
| --- | --- |
| #1 | "Carcinoma, Hepatocellular"[Mesh] |
| #2 | (((((((((((((((((((((((((Carcinoma, Hepatocellular[Title/Abstract]) OR (Hepatocellular Carcinomas[Title/Abstract])) OR (Hepatocellular Carcinoma[Title/Abstract])) OR (Hepatoma[Title/Abstract])) OR (Hepatomas[Title/Abstract])) OR (Liver Cell Carcinoma[Title/Abstract])) OR (Liver Cell Carcinomas[Title/Abstract])) OR (Liver Neoplasms[Title/Abstract])) OR (Hepatic Neoplasms[Title/Abstract])) OR (Hepatic Neoplasm[Title/Abstract])) OR (Liver Neoplasm[Title/Abstract])) OR (Liver Cancer[Title/Abstract])) OR (Liver Cancers[Title/Abstract])) OR (Hepatocellular Cancer[Title/Abstract])) OR (Hepatocellular Cancers[Title/Abstract])) OR (Hepatic Cancer[Title/Abstract])) OR (Hepatic Cancers[Title/Abstract])) OR (Liver Cell Adenoma[Title/Abstract])) OR (Liver Cell Adenomas[Title/Abstract])) OR (Hepatocellular Adenoma[Title/Abstract])) OR (Hepatocellular Adenomas[Title/Abstract])) OR (Benign Hepatoma[Title/Abstract])) OR (Benign Hepatomas[Title/Abstract])) OR (hepatic cell carcinoma[Title/Abstract])) OR (hepatic malignancies[Title/Abstract])) OR (hepatic malignancy[Title/Abstract]) |
| #3 | ("Carcinoma, Hepatocellular"[Mesh]) OR ((((((((((((((((((((((((((Carcinoma, Hepatocellular[Title/Abstract]) OR (Hepatocellular Carcinomas[Title/Abstract])) OR (Hepatocellular Carcinoma[Title/Abstract])) OR (Hepatoma[Title/Abstract])) OR (Hepatomas[Title/Abstract])) OR (Liver Cell Carcinoma[Title/Abstract])) OR (Liver Cell Carcinomas[Title/Abstract])) OR (Liver Neoplasms[Title/Abstract])) OR (Hepatic Neoplasms[Title/Abstract])) OR (Hepatic Neoplasm[Title/Abstract])) OR (Liver Neoplasm[Title/Abstract])) OR (Liver Cancer[Title/Abstract])) OR (Liver Cancers[Title/Abstract])) OR (Hepatocellular Cancer[Title/Abstract])) OR (Hepatocellular Cancers[Title/Abstract])) OR (Hepatic Cancer[Title/Abstract])) OR (Hepatic Cancers[Title/Abstract])) OR (Liver Cell Adenoma[Title/Abstract])) OR (Liver Cell Adenomas[Title/Abstract])) OR (Hepatocellular Adenoma[Title/Abstract])) OR (Hepatocellular Adenomas[Title/Abstract])) OR (Benign Hepatoma[Title/Abstract])) OR (Benign Hepatomas[Title/Abstract])) OR (hepatic cell carcinoma[Title/Abstract])) OR (hepatic malignancies[Title/Abstract])) OR (hepatic malignancy[Title/Abstract])) |
| #4 | deep learning[MeSH Terms] |
| #5 | (((((((((((((((((((((((((((((((Neural Networks[Title/Abstract]) OR (deep learning[Title/Abstract])) OR (Neural Network[Title/Abstract])) OR (CNN[Title/Abstract])) OR (AlexNet[Title/Abstract])) OR (VGGNet[Title/Abstract])) OR (ResNet[Title/Abstract])) OR (GoogLeNet[Title/Abstract])) OR (DenseNet[Title/Abstract])) OR (MobileNet[Title/Abstract])) OR (EfficientNet[Title/Abstract])) OR (VGG-11[Title/Abstract])) OR (VGG-13[Title/Abstract])) OR (VGG-16[Title/Abstract])) OR (VGG-19[Title/Abstract])) OR (VGG11[Title/Abstract])) OR (VGG13[Title/Abstract])) OR (VGG16[Title/Abstract])) OR (VGG19[Title/Abstract])) OR (ResNet50[Title/Abstract])) OR (ResNet101[Title/Abstract])) OR (ResNet34[Title/Abstract])) OR (ResNet18[Title/Abstract])) OR (Long Short-Term Memory[Title/Abstract])) OR (LSTM[Title/Abstract])) OR (Computer aided diagnostic system[Title/Abstract])) OR (Computer-aided diagnostic system[Title/Abstract])) OR (Transfer Learning[Title/Abstract])) OR (Ensemble Learning[Title/Abstract])) OR (artificial intelligence[Title/Abstract])) OR (Hierarchical Learning[Title/Abstract])) OR (Machine Learning[Title/Abstract]) |
| #6 | (deep learning[MeSH Terms]) OR ((((((((((((((((((((((((((((((((Neural Networks[Title/Abstract]) OR (deep learning[Title/Abstract])) OR (Neural Network[Title/Abstract])) OR (CNN[Title/Abstract])) OR (AlexNet[Title/Abstract])) OR (VGGNet[Title/Abstract])) OR (ResNet[Title/Abstract])) OR (GoogLeNet[Title/Abstract])) OR (DenseNet[Title/Abstract])) OR (MobileNet[Title/Abstract])) OR (EfficientNet[Title/Abstract])) OR (VGG-11[Title/Abstract])) OR (VGG-13[Title/Abstract])) OR (VGG-16[Title/Abstract])) OR (VGG-19[Title/Abstract])) OR (VGG11[Title/Abstract])) OR (VGG13[Title/Abstract])) OR (VGG16[Title/Abstract])) OR (VGG19[Title/Abstract])) OR (ResNet50[Title/Abstract])) OR (ResNet101[Title/Abstract])) OR (ResNet34[Title/Abstract])) OR (ResNet18[Title/Abstract])) OR (Long Short-Term Memory[Title/Abstract])) OR (LSTM[Title/Abstract])) OR (Computer aided diagnostic system[Title/Abstract])) OR (Computer-aided diagnostic system[Title/Abstract])) OR (Transfer Learning[Title/Abstract])) OR (Ensemble Learning[Title/Abstract])) OR (artificial intelligence[Title/Abstract])) OR (Hierarchical Learning[Title/Abstract])) OR (Machine Learning[Title/Abstract])) |
| #7 | (("Carcinoma, Hepatocellular"[Mesh]) OR ((((((((((((((((((((((((((Carcinoma, Hepatocellular[Title/Abstract]) OR (Hepatocellular Carcinomas[Title/Abstract])) OR (Hepatocellular Carcinoma[Title/Abstract])) OR (Hepatoma[Title/Abstract])) OR (Hepatomas[Title/Abstract])) OR (Liver Cell Carcinoma[Title/Abstract])) OR (Liver Cell Carcinomas[Title/Abstract])) OR (Liver Neoplasms[Title/Abstract])) OR (Hepatic Neoplasms[Title/Abstract])) OR (Hepatic Neoplasm[Title/Abstract])) OR (Liver Neoplasm[Title/Abstract])) OR (Liver Cancer[Title/Abstract])) OR (Liver Cancers[Title/Abstract])) OR (Hepatocellular Cancer[Title/Abstract])) OR (Hepatocellular Cancers[Title/Abstract])) OR (Hepatic Cancer[Title/Abstract])) OR (Hepatic Cancers[Title/Abstract])) OR (Liver Cell Adenoma[Title/Abstract])) OR (Liver Cell Adenomas[Title/Abstract])) OR (Hepatocellular Adenoma[Title/Abstract])) OR (Hepatocellular Adenomas[Title/Abstract])) OR (Benign Hepatoma[Title/Abstract])) OR (Benign Hepatomas[Title/Abstract])) OR (hepatic cell carcinoma[Title/Abstract])) OR (hepatic malignancies[Title/Abstract])) OR (hepatic malignancy[Title/Abstract]))) AND ((deep learning[MeSH Terms]) OR ((((((((((((((((((((((((((((((((Neural Networks[Title/Abstract]) OR (deep learning[Title/Abstract])) OR (Neural Network[Title/Abstract])) OR (CNN[Title/Abstract])) OR (AlexNet[Title/Abstract])) OR (VGGNet[Title/Abstract])) OR (ResNet[Title/Abstract])) OR (GoogLeNet[Title/Abstract])) OR (DenseNet[Title/Abstract])) OR (MobileNet[Title/Abstract])) OR (EfficientNet[Title/Abstract])) OR (VGG-11[Title/Abstract])) OR (VGG-13[Title/Abstract])) OR (VGG-16[Title/Abstract])) OR (VGG-19[Title/Abstract])) OR (VGG11[Title/Abstract])) OR (VGG13[Title/Abstract])) OR (VGG16[Title/Abstract])) OR (VGG19[Title/Abstract])) OR (ResNet50[Title/Abstract])) OR (ResNet101[Title/Abstract])) OR (ResNet34[Title/Abstract])) OR (ResNet18[Title/Abstract])) OR (Long Short-Term Memory[Title/Abstract])) OR (LSTM[Title/Abstract])) OR (Computer aided diagnostic system[Title/Abstract])) OR (Computer-aided diagnostic system[Title/Abstract])) OR (Transfer Learning[Title/Abstract])) OR (Ensemble Learning[Title/Abstract])) OR (artificial intelligence[Title/Abstract])) OR (Hierarchical Learning[Title/Abstract])) OR (Machine Learning[Title/Abstract]))) |

**2.Cochrane**

| Search number | Query |
| --- | --- |
| #1 | MeSH descriptor: [Carcinoma, Hepatocellular] explode all trees |
| #2 | (Carcinoma, Hepatocellular):ti,ab,kw OR (Hepatocellular Carcinomas):ti,ab,kw OR (Hepatocellular Carcinoma):ti,ab,kw OR (Hepatoma):ti,ab,kw OR (Hepatomas):ti,ab,kw |
| #3 | (Liver Cell Carcinoma):ti,ab,kw OR (Liver Cell Carcinomas):ti,ab,kw OR (Liver Neoplasms):ti,ab,kw OR (Hepatic Neoplasms):ti,ab,kw OR (Hepatic Neoplasm):ti,ab,kw |
| #4 | (Liver Neoplasm):ti,ab,kw OR (Liver Cancer):ti,ab,kw OR (Liver Cancers):ti,ab,kw OR (Hepatocellular Cancer):ti,ab,kw OR (Hepatocellular Cancers):ti,ab,kw |
| #5 | (Hepatic Cancer):ti,ab,kw OR (Hepatic Cancers):ti,ab,kw OR (Liver Cell Adenoma):ti,ab,kw OR (Liver Cell Adenomas):ti,ab,kw OR (Hepatocellular Adenoma):ti,ab,kw |
| #6 | (Hepatocellular Adenomas):ti,ab,kw OR (Benign Hepatoma):ti,ab,kw OR (Benign Hepatomas):ti,ab,kw OR (hepatic cell carcinoma):ti,ab,kw OR (hepatic malignancies):ti,ab,kw |
| #7 | (hepatic malignancy):ti,ab,kw |
| #8 | #1 or #2 or #3 or #4 or #5 or #6 or #7 |
| #9 | MeSH descriptor: [Deep Learning] explode all trees |
| #10 | (deep learning):ti,ab,kw OR (Neural Networks):ti,ab,kw OR (Neural Network):ti,ab,kw OR (CNN):ti,ab,kw OR (AlexNet):ti,ab,kw |
| #11 | (VGGNet):ti,ab,kw OR (ResNet):ti,ab,kw OR (GoogLeNet):ti,ab,kw OR (DenseNet):ti,ab,kw OR (MobileNet):ti,ab,kw |
| #12 | (EfficientNet):ti,ab,kw OR (VGG-11):ti,ab,kw OR (VGG-13):ti,ab,kw OR (VGG-16):ti,ab,kw OR (VGG-19):ti,ab,kw |
| #13 | (VGG11):ti,ab,kw OR (VGG13):ti,ab,kw OR (VGG16):ti,ab,kw OR (VGG19):ti,ab,kw OR (ResNet50):ti,ab,kw |
| #14 | (ResNet101):ti,ab,kw OR (ResNet34):ti,ab,kw OR (ResNet18):ti,ab,kw OR (Long Short-Term Memory):ti,ab,kw OR (LSTM):ti,ab,kw |
| #15 | (Computer aided diagnostic system):ti,ab,kw OR (Computer-aided diagnostic system):ti,ab,kw OR (Transfer Learning):ti,ab,kw OR (Ensemble Learning):ti,ab,kw OR (artificial intelligence):ti,ab,kw |
| #16 | (Hierarchical Learning):ti,ab,kw OR (Machine Learning):ti,ab,kw |
| #17 | #9 or #10 or #11 or #12 or #13 or #14 or #15 or #16 |
| #18 | #8 and #17 |

**3.Embase**

| Search number | Query |
| --- | --- |
| #1 | 'carcinoma, hepatocellular':ab,ti OR 'hepatocellular carcinomas':ab,ti OR 'hepatocellular carcinoma':ab,ti OR hepatoma:ab,ti OR hepatomas:ab,ti OR 'liver cell carcinoma':ab,ti OR 'liver cell carcinomas':ab,ti OR 'liver neoplasms':ab,ti OR 'hepatic neoplasms':ab,ti OR 'hepatic neoplasm':ab,ti OR 'liver neoplasm':ab,ti OR 'liver cancer':ab,ti OR 'liver cancers':ab,ti OR 'hepatocellular cancer':ab,ti OR 'hepatocellular cancers':ab,ti OR 'hepatic cancer':ab,ti OR 'hepatic cancers':ab,ti OR 'liver cell adenoma':ab,ti OR 'liver cell adenomas':ab,ti OR 'hepatocellular adenoma':ab,ti OR 'hepatocellular adenomas':ab,ti OR 'benign hepatoma':ab,ti OR 'benign hepatomas':ab,ti OR 'hepatic cell carcinoma':ab,ti OR 'hepatic malignancies':ab,ti OR 'hepatic malignancy':ab,ti |
| #2 | 'liver cell carcinoma'/exp |
| #3 | #1 OR #2 |
| #4 | 'deep learning'/exp |
| #5 | 'deep learning':ab,ti OR 'neural networks':ab,ti OR 'neural network':ab,ti OR cnn:ab,ti OR alexnet:ab,ti OR vggnet:ab,ti OR resnet:ab,ti OR googlenet:ab,ti OR densenet:ab,ti OR mobilenet:ab,ti OR efficientnet:ab,ti OR 'vgg 11':ab,ti OR 'vgg 13':ab,ti OR 'vgg 16':ab,ti OR 'vgg 19':ab,ti OR vgg11:ab,ti OR vgg13:ab,ti OR vgg16:ab,ti OR vgg19:ab,ti OR resnet50:ab,ti OR resnet101:ab,ti OR resnet34:ab,ti OR resnet18:ab,ti OR 'long short-term memory':ab,ti OR lstm:ab,ti OR 'computer aided diagnostic system':ab,ti OR 'computer-aided diagnostic system':ab,ti OR 'transfer learning':ab,ti OR 'ensemble learning':ab,ti OR 'artificial intelligence':ab,ti OR 'hierarchical learning':ab,ti OR 'machine learning':ab,ti |
| #6 | #4 OR #5 |
| #7 | #3 AND #6 |

**4.Web of science**

| Search number | Query |
| --- | --- |
| #1 | TS=(Carcinoma, Hepatocellular OR Hepatocellular Carcinomas OR Hepatocellular Carcinoma OR Hepatoma OR Hepatomas OR Liver Cell Carcinoma OR Liver Cell Carcinomas OR Liver Neoplasms OR Hepatic Neoplasms OR Hepatic Neoplasm OR Liver Neoplasm OR Liver Cancer OR Liver Cancers OR Hepatocellular Cancer OR Hepatocellular Cancers OR Hepatic Cancer OR Hepatic Cancers OR Liver Cell Adenoma OR Liver Cell Adenomas OR Hepatocellular Adenoma OR Hepatocellular Adenomas OR Benign Hepatoma OR Benign Hepatomas OR hepatic cell carcinoma OR hepatic malignancies OR hepatic malignancy) |
| #2 | TS=(deep learning OR Neural Networks OR Neural Network OR CNN OR AlexNet OR VGGNet OR ResNet OR GoogLeNet OR DenseNet OR MobileNet OR EfficientNet OR VGG-11 OR VGG-13 OR VGG-16 OR VGG-19 OR VGG11 OR VGG13 OR VGG16 OR VGG19 OR ResNet50 OR ResNet101 OR ResNet34 OR ResNet18 OR Long Short-Term Memory OR LSTM OR Computer aided diagnostic system OR Computer-aided diagnostic system OR Transfer Learning OR Ensemble Learning OR artificial intelligence OR Hierarchical Learning OR Machine Learning) |
| #3 | #1 AND #2 |
