# Supplementary figures and images for: Accuracy of Medical Image–Based Deep Learning for Detecting Microvascular Invasion in Hepatocellular Carcinoma: Systematic Review and Meta-Analysis
Source: J Med Internet Res. 2026 Mar 2;28:e82000. doi: 10.2196/82000 (PMC12954728; doi:10.2196/82000)

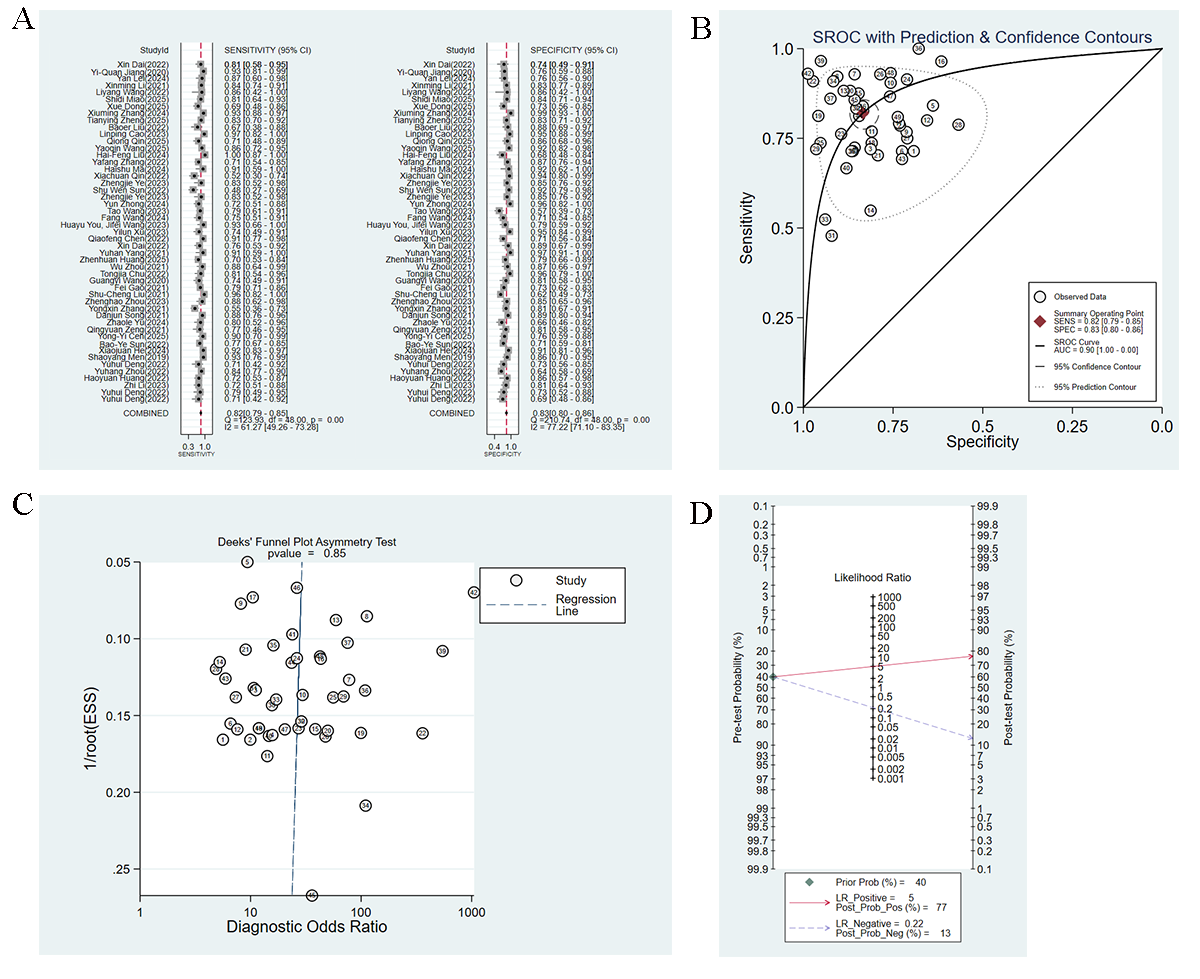

Supplement: Multimedia Appendix 2 [file jmir-v28-e82000-s002.png]

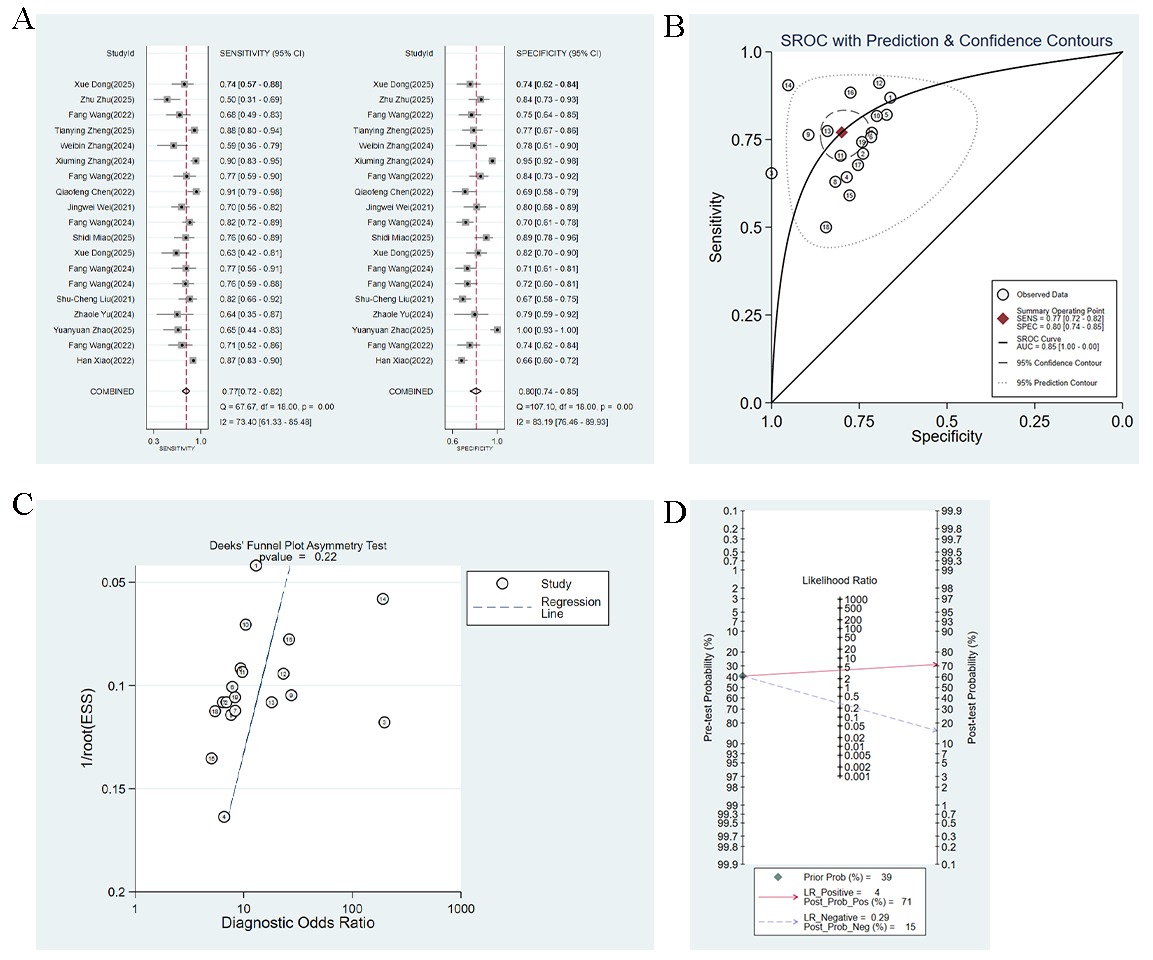

Supplement: Multimedia Appendix 3 [file jmir-v28-e82000-s003.png]

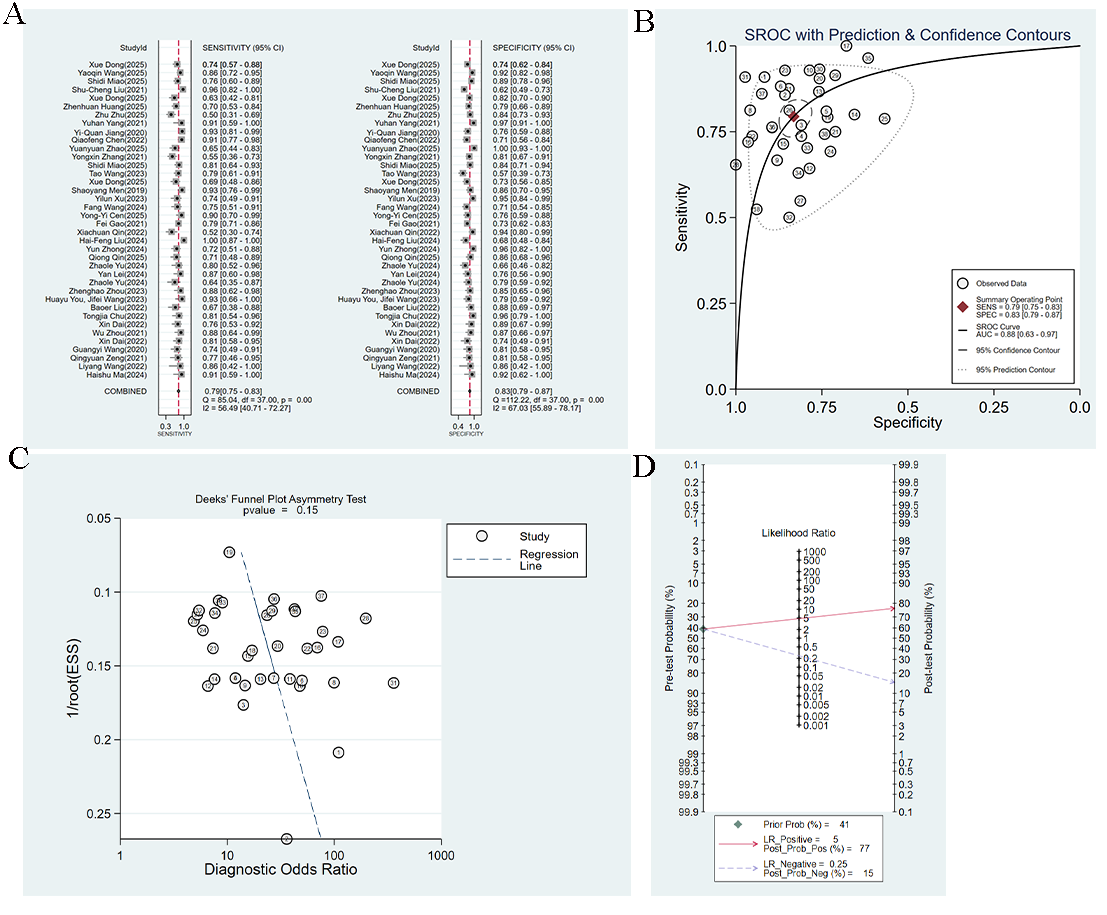

Supplement: Multimedia Appendix 4 [file jmir-v28-e82000-s004.png]

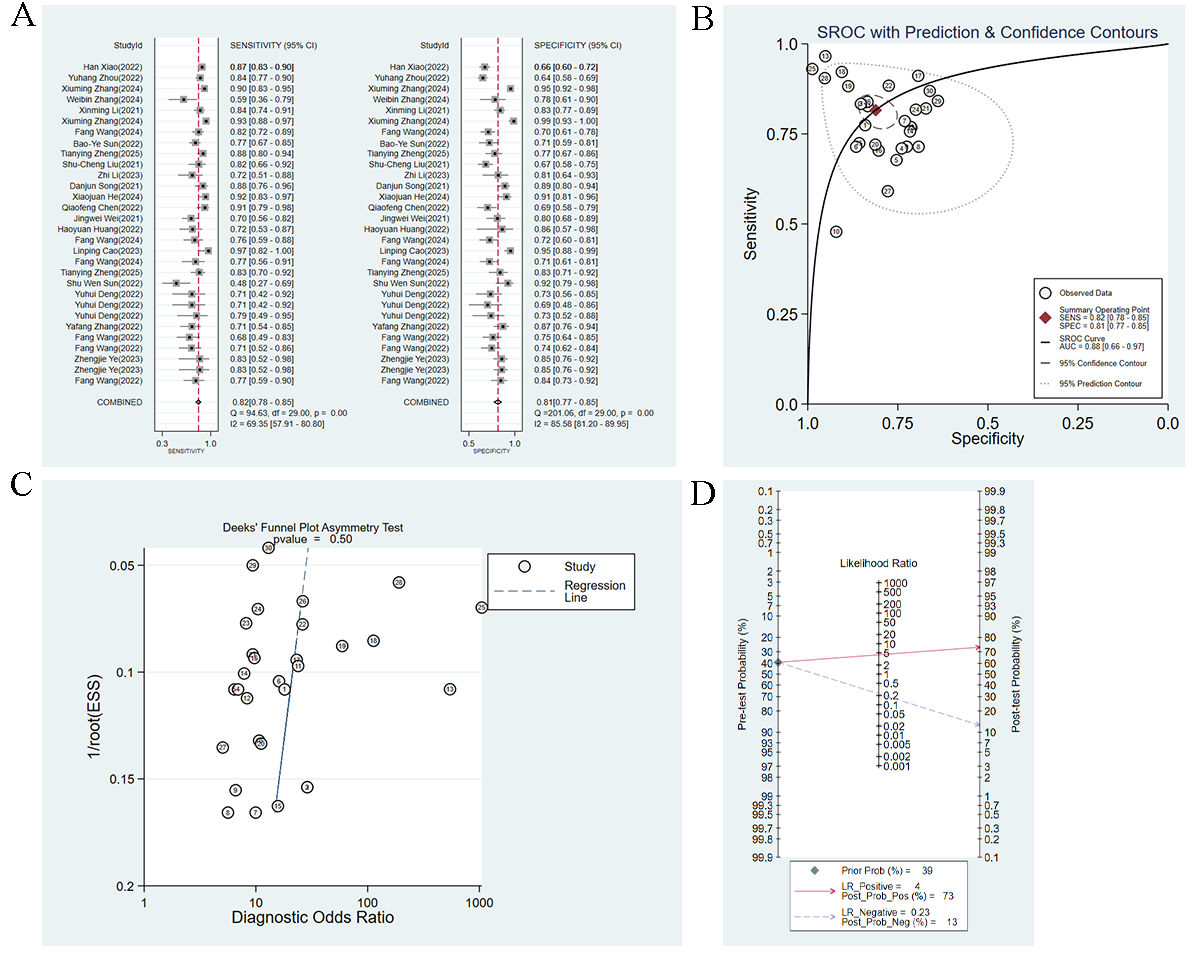

Supplement: Multimedia Appendix 5 [file jmir-v28-e82000-s005.png]

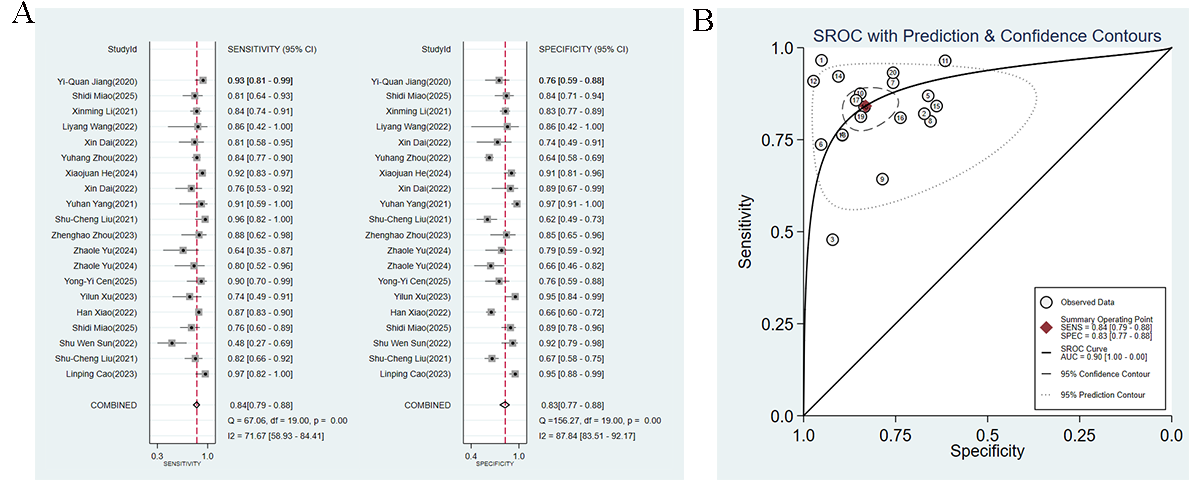

Supplement: Multimedia Appendix 6 [file jmir-v28-e82000-s006.png]

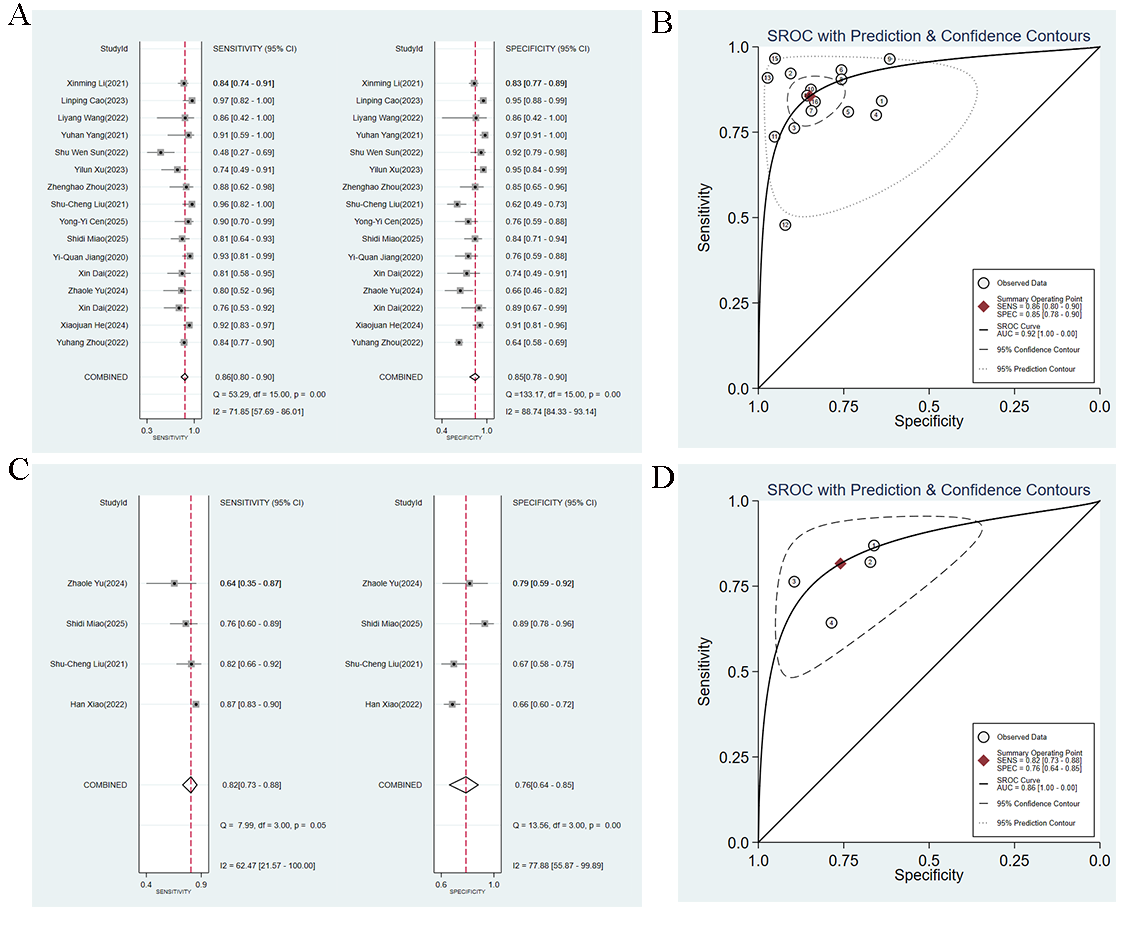

Supplement: Multimedia Appendix 7 [file jmir-v28-e82000-s007.png]

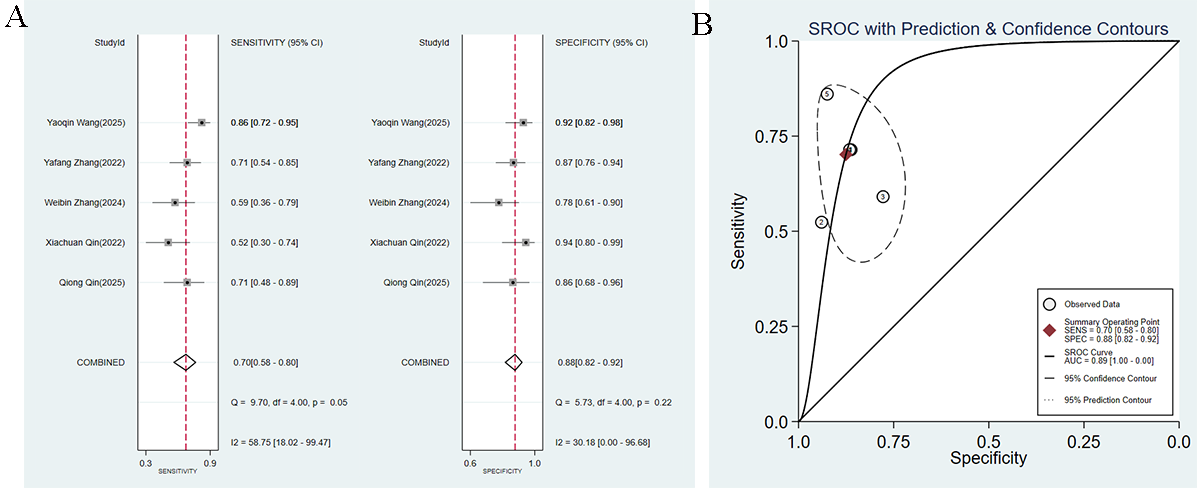

Supplement: Multimedia Appendix 8 [file jmir-v28-e82000-s008.png]

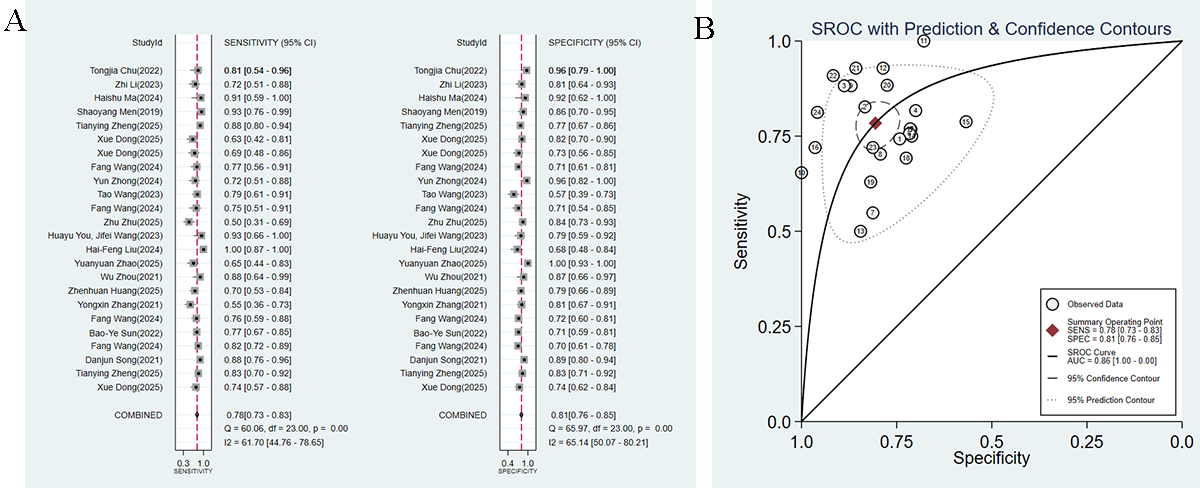

Supplement: Multimedia Appendix 9 [file jmir-v28-e82000-s009.png]

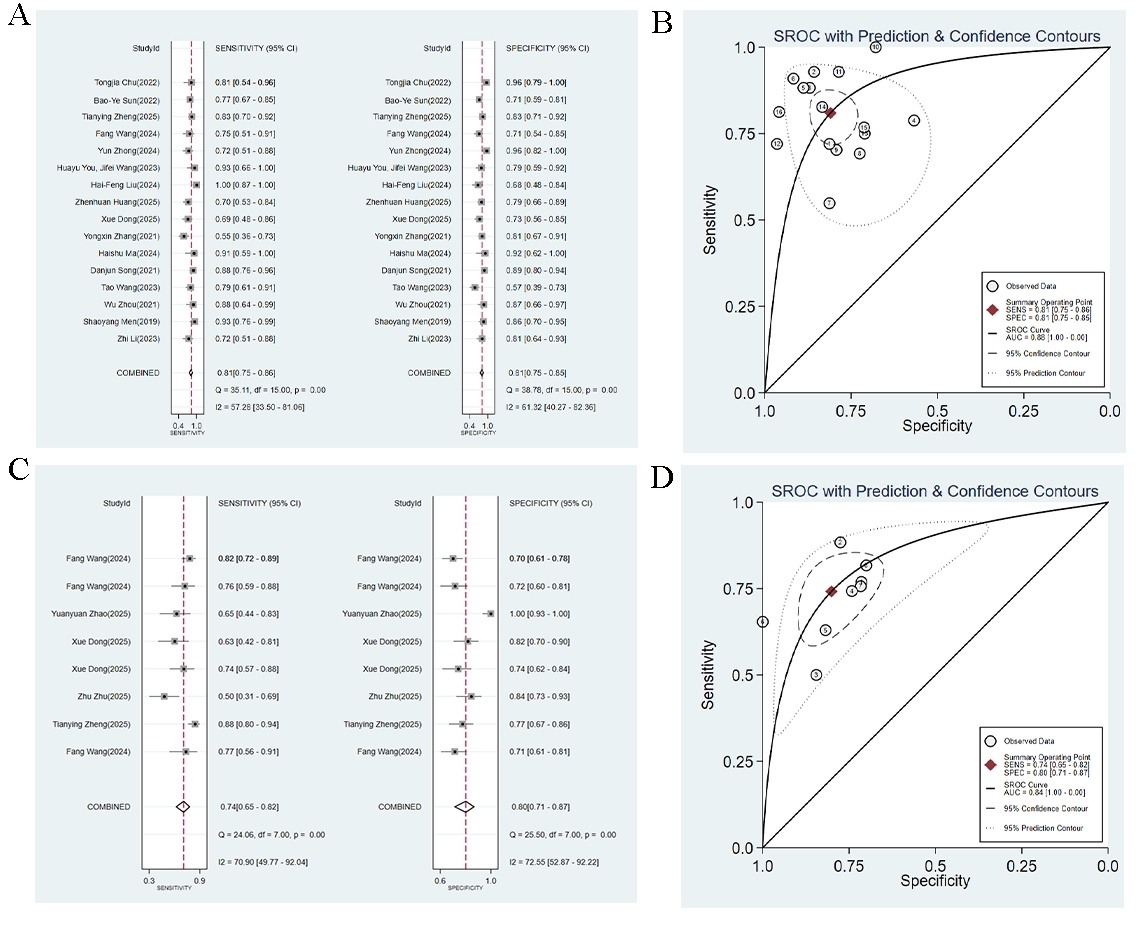

Supplement: Multimedia Appendix 10 [file jmir-v28-e82000-s010.png]

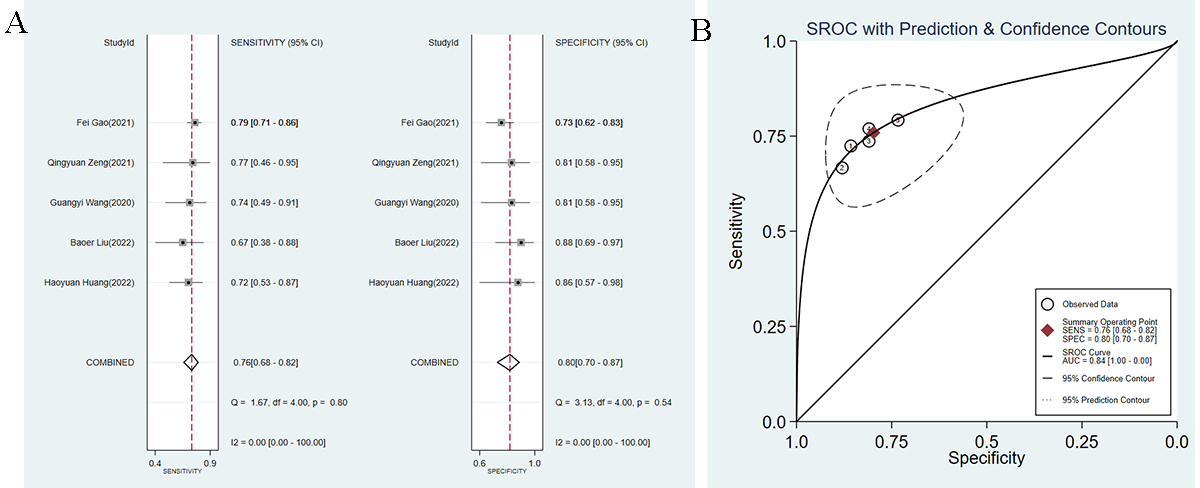

Supplement: Multimedia Appendix 11 [file jmir-v28-e82000-s011.png]

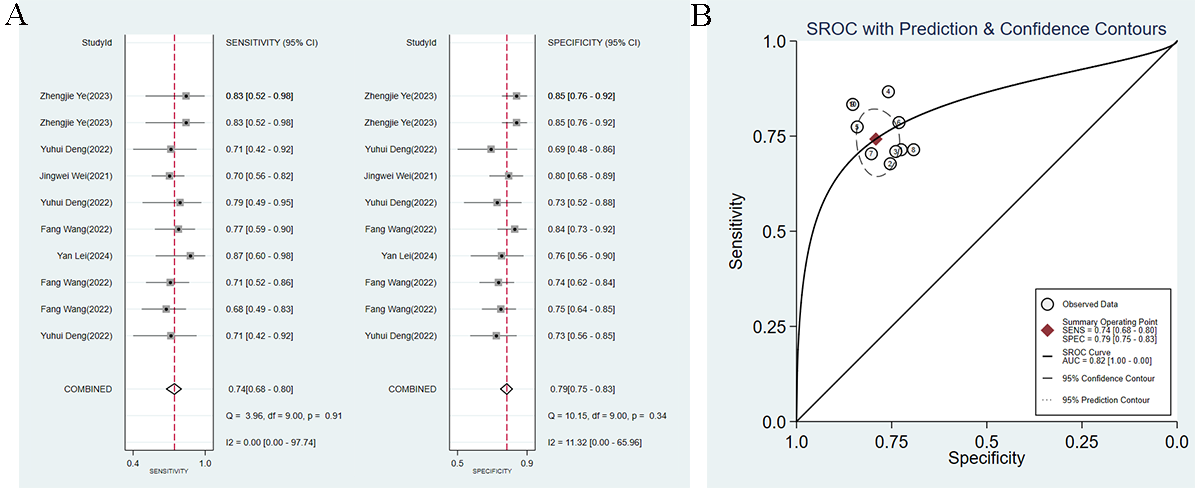

Supplement: Multimedia Appendix 12 [file jmir-v28-e82000-s012.png]

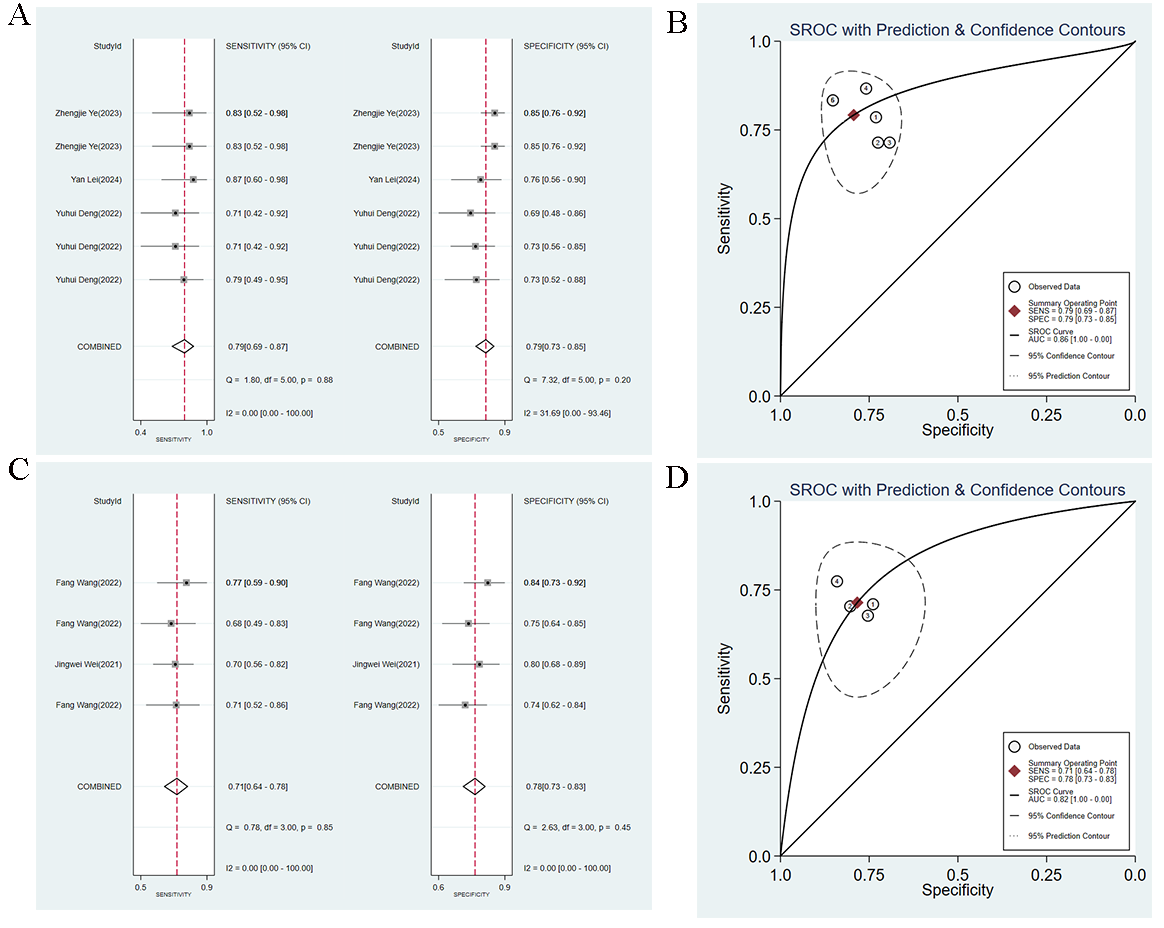

Supplement: Multimedia Appendix 13 [file jmir-v28-e82000-s013.png]

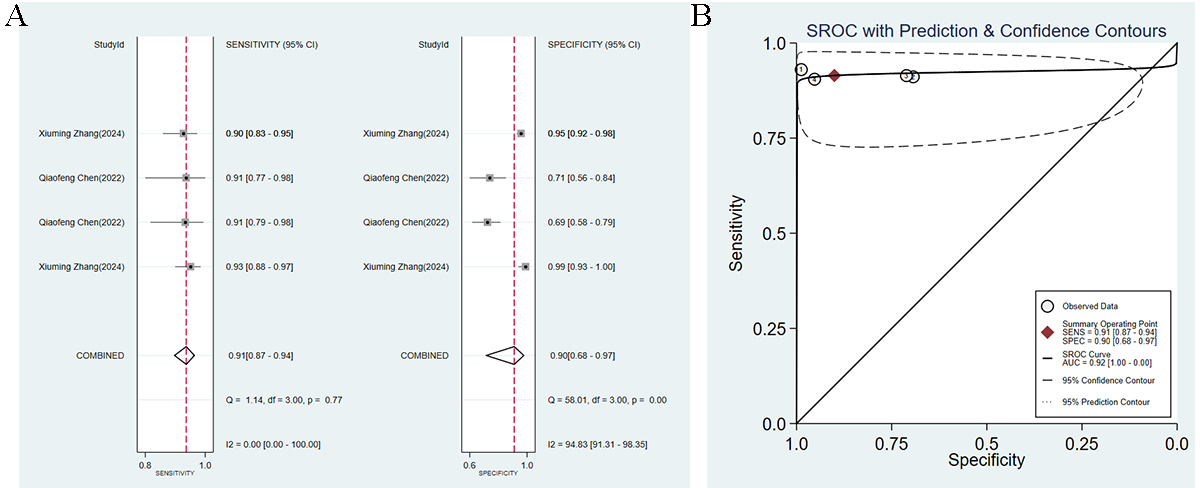

Supplement: Multimedia Appendix 14 [file jmir-v28-e82000-s014.png]
