# Supplementary material for: Accuracy of Medical Image–Based Deep Learning for Detecting Microvascular Invasion in Hepatocellular Carcinoma: Systematic Review and Meta-Analysis
Source: J Med Internet Res. 2026 Mar 2;28:e82000. doi: 10.2196/82000 (PMC12954728; doi:10.2196/82000)
Supplement: Multimedia Appendix 15 [file jmir-v28-e82000-s015.pdf]

No.	Author	Year	Subject selection					Index test (AI)	Reference standard	Workflow
			v1	v2	v3	v4	v5			
1	Xiuming Zhang et al.	2024	Low	High	Low	Low	Low	low	Low	Low
2	Yan Lei et al.	2024	Low	Low	Low	Low	Low	High	Low	Low
3	Hai-Feng Liu et al.	2024	Low	Low	Low	Low	Low	High	Low	Low
4	Zhenghao Zhou et al.	2023	Low	Low	Low	Low	Low	High	Low	Low
5	Xiaojuan He et al.	2024	Low	Low	Low	Low	High	High	Low	Low
6	Yun Zhong et al.	2024	Low	Low	Low	Low	Low	High	Low	Low
7	Fang Wang et al.	2024	Low	Low	Low	Low	Low	low	Low	Low
8	Zhaole Yu et al.	2024	Low	Low	Low	Low	Low	low	Low	Low
9	Haishu Ma et al.	2024	Low	Low	Low	Low	High	High	Low	Low
10	Weibin Zhang et al.	2024	Low	Low	Low	Low	Low	low	Low	Low
11	Fang Wang et al.	2022	Low	Low	Low	Low	High	low	Low	Low
12	Huayu You et al.	2023	Low	Low	Low	Low	Low	High	Low	Low
13	Xiachuan Qin et al.	2022	Low	Low	Low	Low	Low	High	Low	Low
14	Zhi Li et al.	2023	Low	Low	Low	Low	High	High	Low	Low
15	Linping Cao et al.	2023	Low	Low	Low	Low	Low	High	Low	Low
16	Tao Wang et al.	2023	Low	Low	Low	Low	Low	High	Low	Low
17	Zhengjie Ye et al.	2023	Low	Low	Low	Low	Low	High	Low	Low
18	Yilun Xu et al.	2023	Low	Low	Low	Low	Low	High	Low	Low
19	Yuhui Deng et al.	2022	Low	Low	Low	Low	Low	High	Low	Low
20	Xinming Li et al.	2021	Low	Low	Low	Low	Low	High	Low	Low
21	Qiaofeng Chen et al.	2022	Low	Low	Low	Low	Low	low	Low	Low
22	Yafang Zhang et al.	2022	Low	Low	Low	Low	Low	High	Low	Low
23	Baoer Liu et al.	2022	Low	Low	Low	Low	Low	High	Low	Low
24	Shu Wen Sun et al.	2022	Low	Low	Low	Low	Low	High	Low	Low
25	Liyang Wang et al.	2022	Low	Low	Low	Low	Low	High	Low	Low
26	Han Xiao et al.	2022	Low	Low	Low	Low	High	low	Low	Low
27	Yuhan Yang et al.	2021	Low	Low	Low	Low	High	High	Low	Low
28	Bao-Ye Sun et al.	2022	Low	Low	Low	Low	Low	High	Low	Low
29	Xin Dai et al.	2022	Low	Low	Low	Low	High	High	Low	Low
30	Yongxin Zhang et al.	2021	Low	Low	Low	Low	Low	High	Low	Low
31	Shu-Cheng Liu et al.	2021	Low	Low	Low	Low	Low	low	Low	Low
32	Jingwei Wei et al.	2021	Low	Low	Low	Low	Low	low	Low	Low
33	Wu Zhou et al.	2021	Low	Low	Low	Low	Low	High	Low	Low
34	Guangyi Wang et al.	2020	Low	Low	Low	Low	Low	High	Low	Low
35	Qingyuan Zeng et al.	2021	Low	Low	Low	Low	Low	High	Low	Low
36	Fei Gao et al.	2021	Low	Low	Low	Low	Low	High	Low	Low
37	Yi-Quan Jiang et al.	2020	Low	Low	Low	Low	Low	High	Low	Low
38	Danjun Song et al.	2021	Low	Low	Low	Low	Low	High	Low	Low
39	Shaoyang Men et al.	2019	Low	Low	Low	Low	Low	High	Low	Low
40	Yuhang Zhou et al.	2022	Low	Low	Low	Low	Low	High	Low	Low
41	Tongjia Chu et al.	2022	Low	Low	Low	Low	Low	High	Low	Low
42	Haoyuan Huang et al.	2022	Low	Low	Low	Low	Low	High	Low	Low
43	Yaoqin Wang et al.	2025	Low	Low	Low	Low	Low	High	Low	Low
44	Yong-Yi Cen et al.	2025	Low	Low	Low	Low	Low	High	Low	Low
45	Zhenhuan Huang et al.	2025	Low	Low	Low	Low	Low	High	Low	Low
46	Shidi Miao et al.	2025	Low	Low	Low	Low	High	low	Low	Low
47	Zhu Zhu et al.	2025	Low	Low	Low	Low	Low	low	Low	Low
48	Xue Dong et al.	2025	Low	Low	Low	Low	High	low	Low	Low
49	Yinghao Zhang et al.	2024	Low	Low	Low	Low	High	High	Low	Low
50	Tianying Zheng et al.	2025	Low	Low	Low	Low	High	low	Low	Low
51	Yuanyuan Zhao et al.	2025	Low	Low	Low	Low	Low	low	Low	Low
52	Qiong Qin et al.	2025	Low	Low	Low	Low	Low	High	Low	Low

References

1. Zhang X, Yu X, Liang W, Zhang Z, Zhang S, Xu L, et al. Deep learning-based accurate diagnosis and quantitative evaluation of microvascular invasion in hepatocellular carcinoma on whole-slide histopathology images. Cancer Med. 2024 Mar;13(5): e7104. PMID: 38488408. doi: 10.1002/cam4.7104.

2. Lei Y, Feng B, Wan M, Xu K, Cui J, Ma C, et al. Predicting microvascular invasion in hepatocellular carcinoma with a CT- and MRI-based multimodal deep learning model. Abdom Radiol (NY). 2024 May;49(5):1397-410. PMID: 38433144. doi: 10.1007/s00261-024-04202-1.

3. Liu HF, Wang M, Lu YJ, Wang Q, Lu Y, Xing F, et al. CEMRI-Based Quantification of Intratumoral Heterogeneity for Predicting Aggressive Characteristics of Hepatocellular Carcinoma Using Habitat Analysis: Comparison and Combination of Deep Learning. Acad Radiol. 2024 Jun;31(6):2346-55. PMID: 38057182. doi: 10.1016/j.acra.2023.11.024.

4. Zhou Z, Xia T, Zhang T, Du M, Zhong J, Huang Y, et al. Prediction of preoperative microvascular invasion by dynamic radiomic analysis based on contrast-enhanced computed tomography. Abdom Radiol (NY). 2024 Feb;49(2):611-24. PMID: 38051358. doi: 10.1007/s00261-023-04102-w.

5. He X, Xu Y, Zhou C, Song R, Liu Y, Zhang H, et al. Prediction of microvascular invasion and pathological differentiation of hepatocellular carcinoma based on a deep learning model. Eur J Radiol. 2024 Mar;172:111348. PMID: 38325190. doi: 10.1016/j.ejrad.2024.111348.

6. Zhong Y, Chen L, Ding F, Ou W, Zhang X, Weng S. Assessing microvascular invasion in HBV-related hepatocellular carcinoma: an online interactive nomogram integrating inflammatory markers, radiomics, and convolutional neural networks. Front Oncol. 2024;14:1401095. PMID: 39351352. doi: 10.3389/fonc.2024.1401095.

7. Wang F, Zhan G, Chen QQ, Xu HY, Cao D, Zhang YY, et al. Multitask deep learning for prediction of microvascular invasion and recurrence-free survival in hepatocellular carcinoma based on MRI images. Liver Int. 2024 Jun;44(6):1351-62. PMID: 38436551. doi: 10.1111/liv.15870.

8. Yu Z, Liu Y, Dai X, Cui E, Cui J, Ma C. Enhancing preoperative diagnosis of microvascular

invasion in hepatocellular carcinoma: domain-adaptation fusion of multi-phase CT images. Front Oncol. 2024; 14:1332188. PMID: 38333689. doi: 10.3389/fonc.2024.1332188.

9. Ma H, Wang L, Sun L, Wang S, Lu L, Zhang C, et al. Preoperative Prediction of Microvascular Invasion in Hepatocellular Carcinoma From Multi-Sequence Magnetic Resonance Imaging Based on Deep Fusion Representation Learning. IEEE J Biomed Health Inform. 2025 May;29(5):3259-71. PMID: 39196745. doi: 10.1109/jbhi.2024.3451331.

10. Zhang W, Guo Q, Zhu Y, Wang M, Zhang T, Cheng G, et al. Cross-institutional evaluation of deep learning and radiomics models in predicting microvascular invasion in hepatocellular carcinoma: validity, robustness, and ultrasound modality efficacy comparison. Cancer Imaging. 2024 Oct 22;24(1):142. PMID: 39438929. doi: 10.1186/s40644-024-00790-9.

11. Wang F, Chen Q, Chen Y, Zhu Y, Zhang Y, Cao D, et al. A novel multimodal deep learning model for preoperative prediction of microvascular invasion and outcome in hepatocellular carcinoma. Eur J Surg Oncol. 2023 Jan;49(1):156-64. PMID: 36333180. doi: 10.1016/j.ejso.2022.08.036.

12. You H, Wang J, Ma R, Chen Y, Li L, Song C, et al. Clinical Interpretability of Deep Learning for Predicting Microvascular Invasion in Hepatocellular Carcinoma by Using Attention Mechanism. Bioengineering (Basel). 2023 Aug 9;10(8). PMID: 37627833. doi: 10.3390/bioengineering10080948.

13. Qin X, Zhu J, Tu Z, Ma Q, Tang J, Zhang C. Contrast-Enhanced Ultrasound with Deep Learning with Attention Mechanisms for Predicting Microvascular Invasion in Single Hepatocellular Carcinoma. Acad Radiol. 2023 Sep;30 Suppl 1:S73-s80. PMID: 36567144. doi: 10.1016/j.acra.2022.12.005.

14. Li Z, Wang Y, Zhu Y, Xu J, Wei J, Xie J, et al. Modality-based attention and dual-stream multiple instance convolutional neural network for predicting microvascular invasion of hepatocellular carcinoma. Front Oncol. 2023;13:1195110. PMID: 37434971. doi: 10.3389/fonc.2023.1195110.

15. Cao L, Wang Q, Hong J, Han Y, Zhang W, Zhong X, et al. MVI-TR: A Transformer-Based

Deep Learning Model with Contrast-Enhanced CT for Preoperative Prediction of Microvascular Invasion in Hepatocellular Carcinoma. Cancers (Basel). 2023 Feb 28;15(5). PMID: 36900327. doi: 10.3390/cancers15051538.

16. Wang T, Li Z, Yu H, Duan C, Feng W, Chang L, et al. Prediction of microvascular invasion in hepatocellular carcinoma based on preoperative Gd-EOB-DTPA-enhanced MRI: Comparison of predictive performance among 2D, 2D-expansion and 3D deep learning models. Front Oncol. 2023;13:987781. PMID: 36816963. doi: 10.3389/fonc.2023.987781.

17. Ye Z, Zhang J, Wu N, Chen S, Wang Y, Yu Z, et al. PET-guided attention for prediction of microvascular invasion in preoperative hepatocellular carcinoma on PET/CT. Ann Nucl Med. 2023 Apr;37(4):238-45. PMID: 36723705. doi: 10.1007/s12149-023-01822-3.

18. Xu Y, Chen Y, Wu J, Pan J, Liao C, Su H. The utility of Vision Transformer in preoperatively predicting microvascular invasion status of hepatocellular carcinoma. HPB (Oxford). 2023 May;25(5):533-42. PMID: 36801198. doi: 10.1016/j.hpb.2023.01.015.

19. Deng Y, Jia X, Yu G, Hou J, Xu H, Ren A, et al. Can a proposed double branch multimodality-contribution-aware TripNet improve the prediction performance of the microvascular invasion of hepatocellular carcinoma based on small samples? Front Oncol. 2022;12:1035775. PMID: 36387069. doi: 10.3389/fonc.2022.1035775.

20. Li X, Qi Z, Du H, Geng Z, Li Z, Qin S, et al. Deep convolutional neural network for preoperative prediction of microvascular invasion and clinical outcomes in patients with HCCs. Eur Radiol. 2022 Feb;32(2):771-82. PMID: 34347160. doi: 10.1007/s00330-021-08198-w.

21. Chen Q, Xiao H, Gu Y, Weng Z, Wei L, Li B, et al. Deep learning for evaluation of microvascular invasion in hepatocellular carcinoma from tumor areas of histology images. Hepatol Int. 2022 Jun;16(3):590-602. PMID: 35349075. doi: 10.1007/s12072-022-10323-w.

22. Zhang Y, Wei Q, Huang Y, Yao Z, Yan C, Zou X, et al. Deep Learning of Liver Contrast-Enhanced Ultrasound to Predict Microvascular Invasion and Prognosis in Hepatocellular Carcinoma. Front Oncol. 2022;12:878061. PMID: 35875110. doi: 10.3389/fonc.2022.878061.

23. Liu B, Zeng Q, Huang J, Zhang J, Zheng Z, Liao Y, et al. IVIM using convolutional neural networks predicts microvascular invasion in HCC. Eur Radiol. 2022 Oct;32(10):7185-95. PMID: 35713662. doi: 10.1007/s00330-022-08927-9.

24. Sun SW, Xu X, Liu QP, Chen JN, Zhu FP, Liu XS, et al. LiSNet: An artificial intelligence -based tool for liver imaging staging of hepatocellular carcinoma aggressiveness. Med Phys. 2022 Nov;49(11):6903-13. PMID: 36134900. doi: 10.1002/mp.15972.

25. Wang L, Wu M, Li R, Xu X, Zhu C, Feng X. MVI-Mind: A Novel Deep-Learning Strategy Using Computed Tomography (CT)-Based Radiomics for End-to-End High Efficiency Prediction of Microvascular Invasion in Hepatocellular Carcinoma. Cancers (Basel). 2022 Jun 15;14(12). PMID: 35740620. doi: 10.3390/cancers14122956.

26. Xiao H, Guo Y, Zhou Q, Chen Q, Du Q, Chen S, et al. Prediction of microvascular invasion in hepatocellular carcinoma with expert-inspiration and skeleton sharing deep learning. Liver Int. 2022 Jun;42(6):1423-31. PMID: 35319151. doi: 10.1111/liv.15254.

27. Yang Y, Zhou Y, Zhou C, Ma X. Deep learning radiomics based on contrast enhanced computed tomography predicts microvascular invasion and survival outcome in early stage hepatocellular carcinoma. Eur J Surg Oncol. 2022 May;48(5):1068-77. PMID: 34862094. doi: 10.1016/j.ejso.2021.11.120.

28. Sun BY, Gu PY, Guan RY, Zhou C, Lu JW, Yang ZF, et al. Deep-learning-based analysis of preoperative MRI predicts microvascular invasion and outcome in hepatocellular carcinoma. World J Surg Oncol. 2022 Jun 8;20(1):189. PMID: 35676669. doi: 10.1186/s12957-022-02645-8.

29. Dai X, Cao S, Guo Y, Shi W, Zhang L, Li M, et al., editors. Preoperative Identification of Microvascular Invasion in Hepatocellular Carcinoma based on Multi-modal and Multi response Convolutional Neural Network. Fourteenth International Conference on Graphics and Image Processing; 2022.

30. Zhang Y, Lv X, Qiu J, Zhang B, Zhang L, Fang J, et al. Deep Learning With 3D Convolutional Neural Network for Noninvasive Prediction of Microvascular Invasion in Hepatocellular Carcinoma. Journal of Magnetic Resonance Imaging. 2021;54(1):134-43. doi: 10.1002/jmri.27538.

31. Liu SC, Lai J, Huang JY, Cho CF, Lee PH, Lu MH, et al. Predicting microvascular invasion in hepatocellular carcinoma: a deep learning model validated across hospitals. Cancer Imaging. 2021 Oct 9;21(1):56. PMID: 34627393. doi: 10.1186/s40644-021-00425-3.

32. Wei J, Jiang H, Zeng M, Wang M, Niu M, Gu D, et al. Prediction of Microvascular Invasion in Hepatocellular Carcinoma via Deep Learning: A Multi-Center and Prospective Validation Study. Cancers (Basel). 2021 May 14;13(10). PMID: 34068972. doi: 10.3390/cancers13102368.

33. Zhou W, Jian W, Cen X, Zhang L, Guo H, Liu Z, et al. Prediction of Microvascular Invasion of Hepatocellular Carcinoma Based on Contrast-Enhanced MR and 3D Convolutional Neural Networks. Front Oncol. 2021; 11: 588010. PMID: 33854959. doi: 10.3389/fonc.2021.588010.

34. Wang G, Jian W, Cen X, Zhang L, Guo H, Liu Z, et al. Prediction of Microvascular Invasion of Hepatocellular Carcinoma Based on Preoperative Diffusion-Weighted MR Using Deep Learning. Acad Radiol. 2021 Nov;28 Suppl 1: S118-s27. PMID: 33303346. doi: 10.1016/j.acra.2020.11.014.

35. Zeng Q, Liu B, Xu Y, Zhou W. An attention-based deep learning model for predicting microvascular invasion of hepatocellular carcinoma using an intra-voxel incoherent motion model of diffusion-weighted magnetic resonance imaging. Phys Med Biol. 2021 Sep 17;66(18). PMID: 34469880. doi: 10.1088/1361-6560/ac22db.

36. Gao F, Qiao K, Yan B, Wu M, Wang L, Chen J, et al. Hybrid network with difference degree and attention mechanism combined with radiomics (H-DARnet) for MVI prediction in HCC. Magn Reson Imaging. 2021 Nov; 83: 27-40. PMID: 34147593. doi: 10.1016/j.mri.2021.06.018.

37. Jiang YQ, Cao SE, Cao S, Chen JN, Wang GY, Shi WQ, et al. Preoperative identification of microvascular invasion in hepatocellular carcinoma by XGBoost and deep learning. J Cancer Res Clin Oncol. 2021 Mar;147(3):821-33. PMID: 32852634. doi: 10.1007/s00432-020-03366-9.

38. Song D, Wang Y, Wang W, Wang Y, Cai J, Zhu K, et al. Using deep learning to predict microvascular invasion in hepatocellular carcinoma based on dynamic contrast-enhanced

MRI combined with clinical parameters. J Cancer Res Clin Oncol. 2021 Dec;147(12):3757-67. PMID: 33839938. doi: 10.1007/s00432-021-03617-3.

39. Men h, Ju H, Zhang L, Zhou W, editors. PREDICTION OF MICROVASCULAR INVASION OF HEPATOCELLULAR CARCINOMA WITH CONTRAST-ENHANCED MR USING 3D CNN AND LSTM. 2019 IEEE 16th International Symposium on Biomedical Imaging; 2019; Venice.

40. Zhou Y, Sun SW, Liu QP, Xu X, Zhang Y, Zhang YD. TED: Two-stage expert-guided interpretable diagnosis framework for microvascular invasion in hepatocellular carcinoma. Med Image Anal. 2022 Nov; 82: 102575. PMID: 36063747. doi: 10.1016/j.media.2022.102575.

41. Chu T, Zhao C, Zhang J, Duan K, Li M, Zhang T, et al. Application of a Convolutional Neural Network for Multitask Learning to Simultaneously Predict Microvascular Invasion and Vessels that Encapsulate Tumor Clusters in Hepatocellular Carcinoma. Ann Surg Oncol. 2022 Oct;29(11):6774-83. PMID: 35754067. doi: 10.1245/s10434-022-12000-6.

42. Huang H, Liu B, Zhang L, Xu Y, Zhou W, editors. Transformer Based Multi-task Deep Learning with Intravoxel Incoherent Motion Model Fitting for Microvascular Invasion Prediction of Hepatocellular Carcinoma. 2022; Cham: Springer Nature Switzerland.

43. Wang Y, Xie W, Li C, Xu Q, Du Z, Zhong Z, et al. Automated microvascular invasion prediction of hepatocellular carcinoma via deep relation reasoning from dynamic contrast-enhanced ultrasound. Comput Med Imaging Graph. 2025 Sep; 124: 102606. PMID: 40680384. doi: 10.1016/j.compmedimag.2025.102606.

44. Cen YY, Nong HY, Huang XX, Lu XX, Pu CH, Huang LH, et al. Computed tomography-based deep learning and multi-instance learning for predicting microvascular invasion and prognosis in hepatocellular carcinoma. World J Gastroenterol. 2025 Aug 14;31(30):109186. PMID: 40933208. doi: 10.3748/wjg.v31.i30.109186.

45. Huang Z, Huang W, Jiang L, Zheng Y, Pan Y, Yan C, et al. Decision Fusion Model for Predicting Microvascular Invasion in Hepatocellular Carcinoma Based on Multi-MR Habitat Imaging and Machine-Learning Classifiers. Acad Radiol. 2025 Apr;32(4):1971-80. PMID: 39472207. doi: 10.1016/j.acra.2024.10.007.

46. Miao S, Sun M, Li X, Wang M, Jiang Y, Liu Z, et al. Deep Learning-Based Prediction of

Microvascular Invasion and Survival Outcomes in Hepatocellular Carcinoma Using Dual-phase CT Imaging of Tumors and Lesser Omental Adipose: A Multicenter Study. Acad Radiol. 2025 Oct;32(10):5789-801. PMID: 40707265. doi: 10.1016/j.acra.2025.07.015.

47. Zhu Z, Wu K, Lu J, Dai S, Xu D, Fang W, et al. Gd-EOB-DTPA-enhanced MRI radiomics and deep learning models to predict microvascular invasion in hepatocellular carcinoma: a multicenter study. BMC Med Imaging. 2025 Mar 31;25(1):105. PMID: 40165094. doi: 10.1186/s12880-025-01646-9.

48. Dong X, Jia X, Zhang W, Zhang J, Xu H, Xu L, et al. Interpretable and generalizable deep learning model for preoperative assessment of microvascular invasion and outcome in hepatocellular carcinoma based on MRI: a multicenter study. Insights Imaging. 2025 Jul 3;16(1):151. PMID: 40610844. doi: 10.1186/s13244-025-02035-0.

49. Zhang Y, Liu H, Zhu L, Chong H, Fu H, Yu L, et al. Modality-Aware Distillation Network for Microvascular Invasion Prediction of Hepatocellar Carcinoma From MRI Images. IEEE Trans Biomed Eng. 2025 Jun;72(6):1825-36. PMID: 40030752. doi: 10.1109/tbme.2024.3523921.

50. Zheng T, Zhu Y, Jiang H, Yang C, Ye Y, Bashir MR, et al. MRI-Based Topology Deep Learning Model for Noninvasive Prediction of Microvascular Invasion and Assisting Prognostic Stratification in HCC. Liver Int. 2025 Mar;45(3):e16205. PMID: 39992060. doi: 10.1111/liv.16205.

51. Zhao Y, Huang X, Sun M, Chen J, Zhang J, Feng S-t, et al. Predicting microvascular invasion plus cytokeratin 19 expression positivity in hepatocellular carcinoma based on EOB-MRI using multitask deep learning. Hepatoma Res. 2025;10:12. doi: 10.20517/2394-5079.2024.143.

52. Qin Q, Pang J, Li J, Gao R, Wen R, Wu Y, et al. Transformer model based on Sonazoid contrast-enhanced ultrasound for microvascular invasion prediction in hepatocellular carcinoma. Med Phys. 2025 Jul;52(7):e17895. PMID: 40384312. doi: 10.1002/mp.17895.
